# A Rational Design of Isoindigo‐Based Conjugated Microporous *n*‐Type Semiconductors for High Electron Mobility and Conductivity

**DOI:** 10.1002/advs.202303562

**Published:** 2023-08-17

**Authors:** Kayaramkodath Chandran Ranjeesh, Ayman Rezk, Jose Ignacio Martinez, Safa Gaber, Areej Merhi, Tina Skorjanc, Matjaž Finšgar, Gisha Elizabeth Luckachan, Ali Trabolsi, Bilal R. Kaafarani, Ammar Nayfeh, Dinesh Shetty

**Affiliations:** ^1^ Department of Chemistry Khalifa University Abu Dhabi P.O. Box 127788 UAE; ^2^ Department of Electrical Engineering and Computer Science Khalifa University Abu Dhabi P.O. Box 127788 UAE; ^3^ Department of Low‐Dimensional Systems Instituto de Ciencia de Materiales de Madrid‐CSIC C/ Sor Juana Inés de la Cruz 3 Madrid 28049 Spain; ^4^ Department of Chemistry American University of Beirut Beirut 1107‐2020 Lebanon; ^5^ Materials Research Laboratory University of Nova Gorica Vipavska cesta 11c Ajdovscina 5270 Slovenia; ^6^ Faculty of Chemistry and Chemical Engineering University of Maribor Smetanova ulica 17 Maribor 2000 Slovenia; ^7^ Science Division New York University Abu Dhabi Saadiyat Island Abu Dhabi P.O. Box 129188 UAE; ^8^ NYUAD Water Research Center New York University Abu Dhabi (NYUAD) Saadiyat Island Abu Dhabi P.O. Box 129188 UAE; ^9^ Advanced Materials Chemistry Center (AMCC) Khalifa University Abu Dhabi P.O. Box 127788 UAE

**Keywords:** 2D‐polymers, conjugated microporous polymers, electron‐conducting materials, isoindigo, n‐type organic semiconductors

## Abstract

The development of n‐type organic semiconductors has evolved significantly slower in comparison to that of p‐type organic semiconductors mainly due to the lack of electron‐deficient building blocks with stability and processability. However, to realize a variety of organic optoelectronic devices, high‐performance n‐type polymer semiconductors are essential. Herein, conjugated microporous polymers (CMPs) comprising isoindigo acceptor units linked to benzene or pyrene donor units (**BI** and **PI**) showing n‐type semiconducting behavior are reported. In addition, considering the challenges of deposition of a continuous and homogeneous thin film of CMPs for accurate Hall measurements, a plasma‐assisted fabrication technique is developed to yield uniform thin films. The fully conjugated 2D networks in **PI**‐ and **BI**‐CMP films display high electron mobility of 6.6 and 3.5 cm^2^ V^−1^ s^−1^, respectively. The higher carrier concentration in **PI** results in high conductivity (5.3 mS cm^−1^). Both experimental and computational studies are adequately combined to investigate structure–property relations for this intriguing class of materials in the context of organic electronics.

## Graphical abstract

Two n‐type semiconducting conjugated microporous polymers (CMPs) comprising isoindigo acceptor unit with different donor units are developed. A plasma‐assisted fabrication technique is implemented for overcoming the existing fabrication limitations of CMPs to achieve high‐charge carrier mobility.

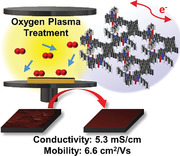

## 1 Introduction

Electron transport is the fundamental process that controls the performance of semiconductors, which are essential components in solar cells, organic light‐emitting diodes (OLEDs), organic field‐effect transistors (OFETs), and thermoelectric generators.^[^ ^1^, ^2^ ^]^ Compared to traditional silicon‐based electronics, organic semiconductors offer unique benefits including low‐temperature solution processability, gravimetric advantage, economy, high flexibility, structural tunability, and diversity.^[^ ^3^, ^4^, ^5^ ^]^ Generally, donor–acceptor (D–A) design is a well‐explored strategy to develop narrow bandgap organic semiconducting materials where an electron‐rich donor component is connected to an electron‐deficient acceptor unit.^[^ ^6^, ^7^ ^]^ However, most of the reported semiconductors exhibit a p‐type character, and therefore, n‐type organic semiconductors evolution is still in its infancy despite the fact that a few n‐type semiconductors have recently shown promising electron mobilities.^[^ ^8^, ^9^, ^10^, ^11^ ^]^

Notably, there are significant obstacles to overcome in order to develop high‐performance n‐type organic semiconductors: i) producing polymers with low‐lying, lowest unoccupied molecular orbital (LUMO) states is a synthetic challenge, ii) a poor electron injection efficiency because of significant energy mismatch between the work function of Au electrodes and the material's LUMO levels, and iii) the relatively poor film‐forming and solution processability of n‐type polymers compared to p‐types.^[^ ^8^, ^9^, ^10^, ^11^ ^]^ Nonetheless, it is required to enhance the performance of both p‐type and n‐type organic semiconductors at the same time to implement diverse organic electronics applications.^[^ ^10^, ^11^, ^12^, ^13^ ^]^ Hence, there is a vital requirement for the development of high‐mobility n‐type organic semiconductors that can perform at ambient conditions.

Over the past few years, much effort has been invested in investigating the nature of the conductivity in conjugated 2D‐porous polymers.^[^ ^14^, ^15^, ^16^, ^17^ ^]^ Despite encouraging mobilities, these polymers were reported to show low conductivities.^[^ ^18^ ^]^ To overcome this challenge, doping techniques have been used to incorporate guest molecules to improve conductivity. Still, this method frequently resulted in irreversible structural changes of the material and nonuniform dopant distribution and leaching.^[^ ^19^, ^20^ ^]^ Conjugated microporous polymers (CMPs) are a unique class of 2D‐materials that combine extensive π‐conjugation with persistently microporous structures that can be utilized in a wide variety of applications.^[^ ^12^ ^]^ However, there has not been much crossover between organic semiconductors and CMP research, although many design factors are similar to those in photocatalysis and photoredox catalysis.^[^ ^12^ ^]^

In this context, we have developed n‐type semiconducting CMPs having an isoindigo acceptor unit with different donor units (benzene and pyrene). Moreover, we have introduced a plasma‐assisted fabrication technique for overcoming the existing fabrication limitations of CMPs due to their insolubility and poor surface adhesion with the substrate^[^ ^12^ ^]^ to prepare 2D‐polymer thin films to perform the conductivity and mobility studies. We have achieved excellent conductivity (5.3 mS cm^−1^) and mobility (6.6 cm^2^ V^−1^ s^−1^) for the optimized donor–acceptor combination pyrene‐isoindigo for **PI** in comparison with benzene‐ isoindigo for **BI** (electron mobility 3.5 cm^2^ V^−1^ s^−1^, conductivity 0.17 mS cm^−1^). The rationale behind choosing the isoindigo acceptor unit in our CMPs is because of its proven excellent properties including low‐lying frontier molecular orbital (FMO) energy levels, backbone planarity and extended conjugation, a large local dipole, good solubility after *N*‐alkylation, and the ease of synthesis on a large scale.^[^ ^8^, ^13^ ^]^ To achieve the optimum stacking and flexibility for the fabrication of the CMPs, we opted to *N*‐alkylate the isoindigo unit with a hexyl side chain.^[^ ^8^, ^13^ ^]^ The current work offers an appealing synthetic chemistry for making 2D‐CMPs with high electron mobility, conductivity, and the useful fabrication of thin films, which has a wide range of potential applications in thin‐film optoelectronic devices. This work sheds light on the fundamental understanding of the donor–acceptor interaction required to elucidate relationships between the structure–electronic properties by using an adequate combination of first‐principles calculations with the deformation potential (DP) formalism, which might facilitate the progress of organic semiconducting materials in new directions.

## 2 Results and Discussion

**Figure 1:**
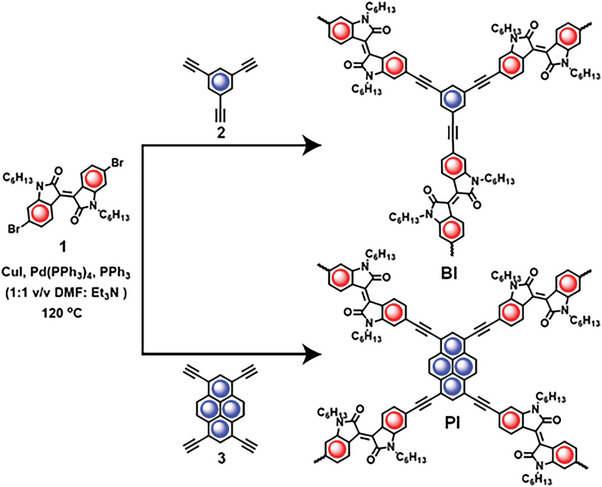
Synthetic scheme for the rationally designed isoindigo‐based n‐type semiconducting conjugated polymers, **BI** and **PI**.

**Figure 2:**
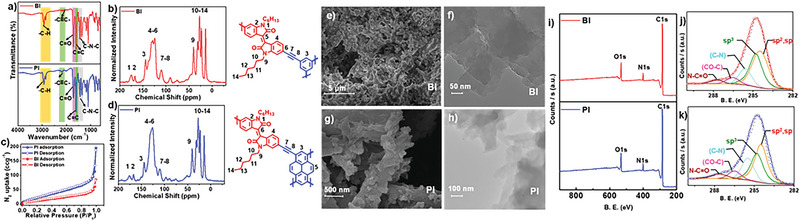
Characterization of CMPs. a) FT‐IR spectra of **BI** and **PI** showing the relevant signals. b,d) ^13^C CP‐MAS NMR spectra of **BI and PI**, respectively. The peaks labeled with numbers and corresponding carbon atoms are shown in the chemical structures. c) N_2_ sorption isotherm of **BI** and **PI** measured at 77 K. SEM images of the e) **BI** and g) **PI**. TEM images of f) **BI** and h) **PI** layered sheets. XPS measurements of **BI** (top) and **PI** (bottom) i) survey spectra, j) C 1s of **BI**, and k) C 1s spectra of **PI**.

Sonogashira–Hagihara coupling reactions were employed to synthesize the polymers (**Figure** **1**) from a dibromo derivative of isoindigo (**1**) acceptor linker and two distinct donor linkers (benzene and pyrene) containing acetylene (**2** and **3**; see the detailed synthetic procedure in the Supporting Information) functional groups. A comparison of the Fourier‐transform infrared (FT‐IR) spectrum of **BI** and **PI** with the starting materials reveals the absence of a C─H band that corresponds to the terminal alkyne at 3,273 cm^−1^, ≡C─H bond stretching of acetylene monomers, and C─Br stretching bands that correspond to the bromo‐isoindigo monomer at 630 cm^−1^ (**Figure** **2**a; and Figure S1, Supporting Information). FT‐IR spectra for both **BI** and **PI** polymer networks exhibit the aromatic C═C vibration bands at 1680 cm^−1^, the alkyne ─C≡C─ stretching band at 2200 cm^−1^, the C─N─C stretching vibration band at 1448 cm^−1^, and the C═O stretching vibrations at 1706 cm^−1^. These observations confirm the successful formation of conjugated networks (Figure 2a; and Figures S1 and S2, Supporting Information). Solid state ^13^carbon cross‐polarization magic‐angle spinning nuclear magnetic resonance (^13^C CP MAS NMR) spectroscopy was also used to characterize the polymer networks at their molecular level. ^13^C CP MAS NMR spectra of **BI** and **PI** (Figure 2b–d) displayed characteristic peaks located at 172 ppm (─**C**═O), 162 ppm (N─**C**), 144–110 ppm (aromatic carbons, ─**C**═**C**─), and 92 ppm (─**C**≡**C**─). These signals originate from the carbon atoms of the isoindigo ring, acetylene linkage, and other aromatic rings within the structure. Moreover, the signals corresponding to the aliphatic hexyl chains are observed at ≈40 ppm (─N─**C**H_2_─), between 30–21 ppm (─**C**H_2_─), and 12 ppm (─**C**H_3_). These NMR spectra provide conclusive evidence for the successful formation of **BI** and **PI** (Figure 2b,d).

Powder X‐ray diffraction (PXRD) patterns of **BI** and **PI** exhibit broad signals, centered at 2θ = 10° and 2θ = 25°, respectively, associated with an amorphous structure of CMPs in the bulk state (Figure S3, Supporting Information). Scanning electron microscope (SEM) images show the bulk structure of **BI** and **PI** composed of stacked layered sheets (Figure 2e,g; and Figures S4 and S5, Supporting Information). Furthermore, the multilayer stacks of the 2D polymers are clearly evident in transmission electron microscopy (TEM) images of **BI** and **PI** (Figure 2f,h; and Figures S6 and S7, Supporting Information). SEM energy dispersive X‐ray (EDX) mapping reveals the elemental composition of **BI** and **PI**, which consists of a uniform distribution of carbon, oxygen, and nitrogen atoms over the entire morphology (Figures S8 and S9, Supporting Information). X‐ray photoelectron spectroscopy (XPS) analysis of **BI** and **PI** clearly showed peaks in C 1s, N 1s, and O 1s spectra, matching the CMP structure, and also confirmed the absence of trapped Pd and Cu catalysts used for the synthesis (Figure 2i,j,k; and Figure S10, Supporting Information). The permanent porosity of **BI** and **PI** was evaluated by measuring N_2_ adsorption isotherms at 77 K. Prior to the BET experiments, **BI** and **PI** were activated for 16 h at 80 °C to release trapped gas molecules and solvents. Reversible type III adsorption isotherms arise from multilayer adsorption for the microporous structures of **BI** and **PI** (Figure 2c).^[^ ^21^ ^]^ The Brunauer–Emmett–Teller (BET) surface areas of the activated **BI** and **PI** samples were found to be 52 and 109 m^2^ g^−1^, respectively. Nonlocal density functional theory reveals a narrow pore size distribution profile centered at 1.8 and 1.9 nm, respectively (Figure S11, Supporting Information). The observed small surface area is plausible because of the strong π–π interactions between the highly conjugated 2D‐layers, which is clearly evident from the broad peak in the PXRD analysis.

**Figure 3:**
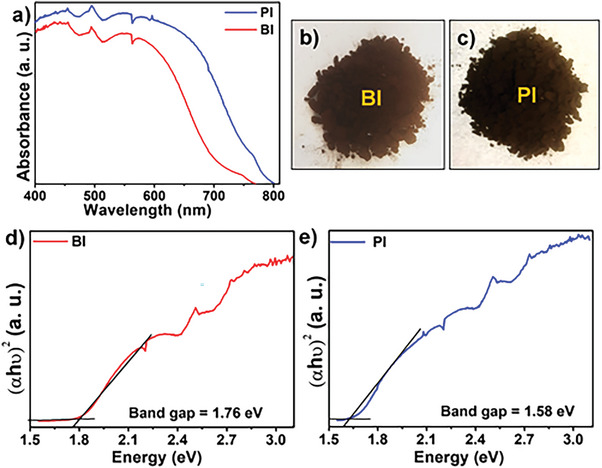
a) Solid‐state UV/VIS absorption spectra of **BI** and **PI**; Visible light photograph of b) **BI** and c) **PI**. Tauc plot of d) **BI** and e) **PI** for bandgap calculations.

The solid‐state ultraviolet/visible diffuse reflectance spectroscopy (UV/VIS DRS) of **BI** and **PI** displayed a broad absorption spectrum with absorption peaks extending up to 750 and 800 nm, respectively (Figure 3a–c). The absorption of **PI** was redshifted by 50 nm compared to **BI** (**Figure** **3**a), showing that the 2D network of **PI** has a longer extended π‐conjugated structure. Both **BI** and **PI** were found to have favorable optical bandgaps of 1.76 and 1.58 eV, respectively, calculated by the Tauc plot method, suggesting the semiconducting nature of these polymers (Figure 3d,e). The effective donor–acceptor interaction between the donor (pyrene) and the acceptor (isoindigo) units reduces the highest‐occupied molecular orbital (HOMO) and LUMO levels in **PI**, leading to a narrowing of the bandgap compared to **BI**. The electrochemical characteristics of **BI** and **PI** were subsequently evaluated using cyclic voltammetry (CV), as illustrated in Figure S12 (Supporting Information). The HOMO levels determined from the oxidation onset of **BI** and **PI** are −5.610 and −5.226 eV, respectively. Their LUMO values are computed from the reduction onset. The LUMO value for **BI** is −3.579, and for **PI** is −3.627 eV, which matches the reported isoindigo‐based n‐type semiconductors.^[^ ^8^, ^13^ ^]^ Table S1 (Supporting Information) summarizes the HOMO/LUMO levels and electrochemical bandgap of **BI** and **PI**. Ultraviolet photoelectron spectroscopy (UPS) was employed to investigate the detailed information on band energy levels of **BI** and **PI**. The calculation of band structure from UPS data implies that Fermi levels are close to the conduction band, and hence they can be potential n‐type materials (Figure S13, Supporting Information).^[^ ^19^ ^]^

**Figure 4:**
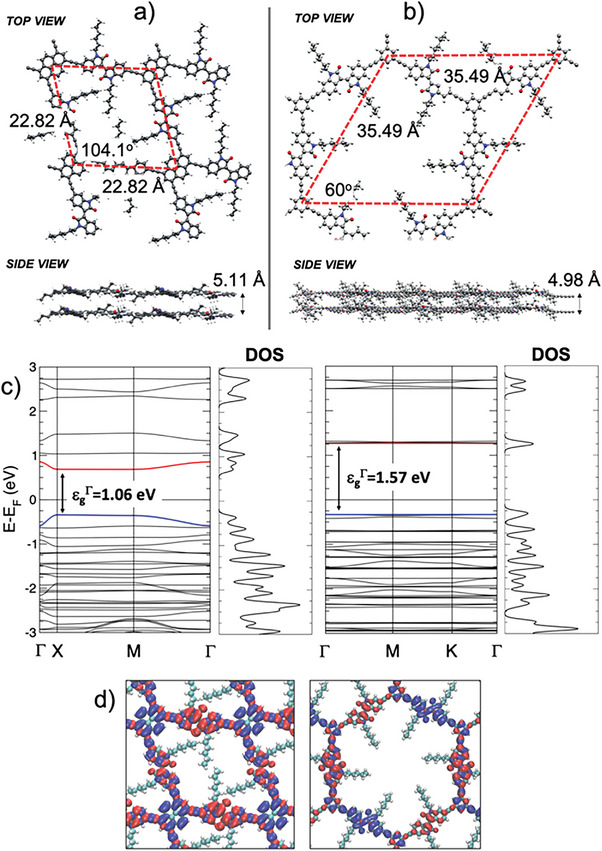
Ground‐state stacking structures of a) **PI** and b) **BI**. Band structures and electronic density of states profiles for **PI** (left) and **BI** (right). d) Electronic orbital diagrams of **PI** (left) and **BI** (right).

With the main goal of obtaining the structural and electronic properties, as well as an estimation of the acoustic‐phonon‐limited electron mobilities, for the **PI** and **BI** compounds, we have carried out a large battery of density functional theory (DFT) based calculations, with an adequate combination with the deformation potential (DP) formalism (see full details in the Supporting Information). Results of the calculations yield a structure for the **PI** compound with a unit cell of [2.28 × 2.28] nm^2^ and an angle between lattice parameters of 104.1^o^, with a preferential stacking configuration very close to the eclipsed (AA) one and an interlayer distance of 5.11 Å, while the **BI** compound exhibits a perfectly hexagonal P6 symmetry with a unit cell of [3.55 × 3.55] nm^2^ and an angle between lattice parameters of 60^o^ (**Figure** **4**a,b). In this case, the preferential stacking fashion results in a perfectly eclipsed AA configuration with an interlayer distance of 4.98 Å (see Figure S14 in the Supporting Information). Regarding their electronic properties, in both cases, they exhibit a clear narrow‐gap semiconducting character with bandgaps at X‐ and Γ‐points of 1.06 and 1.57 eV for the **PI** and **BI**, respectively (see Figure 4c,d), in good agreement with the experimental evidence. On the basis of the outputs obtained from the DFT calculations, we have used the DP formalism to obtain the acoustic‐phonon‐limited electron mobilities at room temperature (300 K), for which we have computed effective electron masses, relaxation times, DP constants which represent the strain‐induced shift of the band edges, and elastic moduli obtained from the lattice distortion by the strain (see full details in the Supporting Information) to finally obtain intrinsic electron mobilities of 44.7 and 8.7 cm^2^ V^−1^ s^−1^, for the **PI** and **BI**, respectively. Despite only adopting the acoustic‐phonon scattering mechanism approximation, together with the fact that the theoretical systems are highly idealized, the comparison between the experimental and theoretical mobilities results is fairly good.

Hall effect measurement is an essential technique to characterize conductivity type, mobility, and carrier density in semiconductive thin films. In light of the first‐principles modeling with the DP formalism, we examined the mobility and conductive properties of both **BI** and **PI** employing the Hall effect under the van der Pauw geometry.^[^ ^22^ ^]^ However, accurate Hall measurements require the deposition of a continuous and homogeneous thin film, especially for CMPs. Therefore, an optimization of the substrate material and film deposition technique was required. The exfoliated **BI** and **PI** are drop‐cast on various 1 × 1 cm^2^ or 3 × 3 cm^2^ pieces of fused silica, quartz, sapphire, and n‐Si with 300 nm of thermal oxide (detailed experimental procedure in the Supporting Information). **BI** and **PI** formed thin films with acceptable homogeneity and uniformity when drop cast on fused silica and thermal oxide substrates, which can be attributed to their noncrystallinity. However, proper surface adhesion remained a continuous challenge even under rapid and gradual thermal annealing on hot plates and ovens for all substrate types. The cross‐linked configuration and nonsolubility in most solvents create a major challenge to form proper thin films from CMPs for incorporation in field effect devices.^[^ ^12^ ^]^ To enhance the adhesion between the polymers and substrates, several established surface alteration methods, such as UV/O_3_, plasma, and corona discharge treatments have been demonstrated.^[^ ^23^ ^]^ We choose to employ plasma treatment on the surface of drop‐casted thin films using a radio frequency (RF) glow discharge in the ambient gas of oxygen. By altering the surface of the substrate using plasma, extra charged electrons are lingering on the surface of the thin film, seeking lower energy states. The surface oxygen plasma activation is optimized in an RIE plasma reactor with 90 W RF power at a 50 sccm flow rate for 3 min. Plasma‐treated films show much better surface adhesion and rigidity than nontreated films, as shown in Figure S15 (Supporting Information).

**Figure 5:**
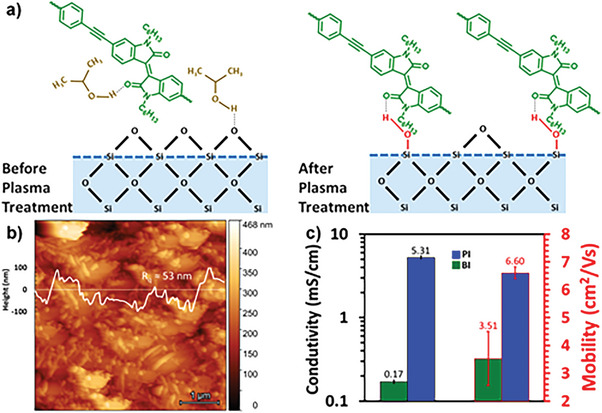
a) Schematics of the drop‐casted 2D‐CMP flakes on the SiO_2_/Si surface without and with oxygen plasma treatment. b) AFM image of the **PI** film. c) The reported mean and std. deviation of the conductivity (left black axis) and mobility (right red axis) for **BI** and **PI** CMPs thin films.

Surface activation triggered by the oxygen plasma treatment results in increased surface reactivity. The activation reaction permits the silanol groups and other functional groups to easily populate the substrate surface, thus improving the 2D‐CMP drop‐casted film and substrate surface adhesion, as shown in **Figure** **5**a.

This type of surface activation reaction of the thermal oxide substrates with oxygen plasma treatment has increased surface hydrophilicity.^[^ ^23^, ^24^ ^]^ The plasma treatment leads to surface cleaning, activation, then chemical modification (hydroxylation) once the silicon oxide surface interacts with the oxygen plasma. The oxygen plasma first cleans the substrate surface by allowing contaminations to be partly removed during bombardment with the oxygen plasma active radicals and particles. The partial decontamination leads to a surface with much more dangling bonds and in turn, elevated surface energy compared to pretreatment surfaces, enhancing surface hydrophilicity. Finally, surface hydroxylation initiates the alteration of the siloxane functional groups (Si─O─Si) to silanol (≡Si─OH) on the substrate surface. Silanol groups can hydrogen bond at room temperature given that they are in the vicinity.^[^ ^25^ ^]^ Directly after the plasma treatment, the exfoliated 2D‐CMPs (**BI** and **PI**) dispersions in isopropyl alcohol (IPA) were drop‐casted onto the pretreated substrates. This rapidly covered the whole surface of the substrate since silanol groups covered the surface of the plasma‐treated substrate and interacted with 2D‐CMPs and IPA molecules via hydrogen bonding rather than Van der Waals forces. The contact between the flakes and the plasma‐treated substrate is boosted by the surface tension throughout the IPA evaporation.^[^ ^26^ ^]^ As a result of the better attraction between the 2D‐CMP thin film and substrate surface, the films have better contact and are more homogenous on the treated surfaces before the drop‐casting procedure, creating continuous and crack‐free films. A representative film of **PI** is shown in an atomic force microscopy (AFM) image (Figure 5b). The absorption features of **BI** and **PI** were compared using UV–VIS spectroscopy and FTIR analysis to investigate the change in optical band gaps and structural changes before and after oxygen plasma treatment‐assisted thin film fabrication. The stability of **BI** and **PI** under tested conditions is confirmed by the absence of considerable changes in spectral and bandgap characteristics from prior experiments (Figures S16 and S17, Supporting Information).

As per the previously mentioned optimized protocol, the exfoliated 2D‐CMPs thin films were prepped for the Hall effect measurements by drop‐casting on 1 × 1 cm^2^ square pieces of thermal oxide wafer. The negative Hall coefficients were found to be consistent with electron conduction and indicated that both **BI** and **PI** are typical n‐type semiconductors. The room temperature Hall carrier densities were ≈5 × 10^15^ and 0.33 × 10^15^ cm^−3^, and thus the implied conductivity were 5.3 and 0.17 mS cm^−1^ for **PI**‐ and **BI**‐CMP films, respectively. The **PI‐** and **BI**‐CMP films displayed a Hall electron mobility in the DC limit of 6.602 ± 0.215 and 3.510 ± 0.964 cm^2^ V^−1^ s^−1^, respectively, as depicted in Figure 5c and **Table** **1**. The E‐k diagrams of **PI** and **BI** show a significant difference in the distribution of both the conduction and valence bands.

**Table 1:** Conductivity, charge carrier concentration, and mobility of **BI** and **PI ‐**CMP thin films (2 samples each) obtained by Hall‐Effect measurements at 25 °C and 0.55 T.

Sample	Conductivity [mS cm^−1^]	Bulk concentration [cm^−3^]	Mobility [cm^2^ V^−1^ s^−1^]
PI_1_	5.332	−5.21 × 10^15^	6.387
PI_2_	5.281	−4.84 × 10^15^	6.817
PI^a)^	5.3065 ± 0.026	(−5.02 ± 0.19) × 10^15^	6.602 ± 0.215
BI_1_	0.1731	−4.238 × 10^14^	2.55
BI_2_	0.1671	−2.33 × 10^14^	4.48
BI^a)^	0.1701 ± 0.003	(−3.284 ± 0.95) × 10^14^	3.51 ± 0.964

^a)^ Mean and std. deviation of the data collected from each sample. All reported data are averaged over 8 concurrent measurements for two samples, considering the roughness and variability of the drop‐casted thin film thicknesses. After each current sweep, samples are then rotated 90°, and the measurement is repeated.

When comparing the valleys of the valence and conduction band, a flatter band shape leads to lower mobility. On the other hand, a more parabolic band shape is expected to have a smaller effective mass and higher carrier mobility. Since the carrier mobility and conductivity are directly proportional to the inverse of the effective mass (1/*m* ^∗^), the observed discrepancy indicates that the measured alteration in mobility can be attributed to a fundamental shift in the band structure. However, it is important to note that our analysis does not account for other scattering mechanisms that may limit mobility. The effective masses calculated near the band edge reveal that the electron/hole effective mass of **PI** along the π‐columns is lower than that of **BI**. This corroborates the Hall effect measurements showing the existence of a greater number of delocalized and mobile carriers in **PI** as compared to **BI**. In short, the Hall electron mobility and conductivity of the **BI** and **PI** thin films were remarkably high and greater than those of the CMPs previously reported (Table S2, Supporting Information).^[^ ^14^, ^15^, ^16^, ^17^ ^]^ Consequently, **PI** exhibits better structural and electronic characteristics that facilitate the transport and segregation of charge carriers. The experimental Hall effect measurement and theoretical mobilities of the DFT results are precisely following the trend.

## 3 Conclusion

In summary, we have demonstrated that the rational design of CMPs with suitable electron donor–acceptor pairs can help to achieve high charge mobility and conductivity in n‐type organic semiconductors. Moreover, our findings suggest that plasma‐assisted fabrication technique is highly useful to prepare uniform thin organic polymer films for the precise Hall effect mobility measurements. The **BI** and **PI** thin films exhibited remarkably high Hall electron mobility and electron conductivity at room temperature, which is higher than that of previously reported CMPs. We also provide reliable structure–electronic property relationship by combining first‐principles modeling with the observed charge‐transport properties. These findings pave the way for the development of efficient conductive n‐type organic semiconductors for future electronic devices.

## Conflict of Interest

The authors declare no conflict of interest.

## Supporting information

Supporting Information

## Data Availability

The data that support the findings of this study are available from the corresponding author upon reasonable request.

## References

[1] H. Bronstein , C. B. Nielsen , B. C. Schroeder , I. McCulloch , Nat. Rev. Chem. 2020, 4, 66. 37128048 10.1038/s41570-019-0152-9

[2] E. Jin , K. Geng , S. Fu , S. Yang , N. Kanlayakan , M. A. Addicoat , N. Kungwan , J. Geurs , H. Xu , M. Bonn , H. I. Wang , J. Smet , T. Kowalczyk , D. Jiang , Chem 2021, 7, 3309.

[3] A. D. Scaccabarozzi , A. Basu , F. Aniés , J. Liu , O. Z. Arteaga , R. Warren , Y. Firdaus , M. I. Nugraha , Y. Lin , M. Campoy‐Quiles , N. Koch , C. Müller , L. Tsetseris , M. Heeney , T. D. Anthopoulos , Chem. Rev. 2022, 122, 4420. 34793134 10.1021/acs.chemrev.1c00581

[4] C. Liao , M. Zhang , M. Y. Yao , T. Hua , L. Li , F. Yan , Adv. Mater. 2015, 27, 7493. 25393596 10.1002/adma.201402625

[5] S. R. Forrest , M. E. Thompson , Chem. Rev. 2007, 107, 923.

[6] J. Zhang , W. Xu , P. Sheng , G. Zhao , D. Zhu , Acc. Chem. Res. 2017, 50, 1654. 28608673 10.1021/acs.accounts.7b00124

[7] a) Y.‐S. Wu , J.‐S. Li , C.‐Y. Chang , W. He , T. Michinobu , Y.‐C. Lin , W.‐C. Chen , C.‐C. Chueh , J. Mater. Chem. C. 2022, 10, 17936.

[8] a) P. Deng , Q. Zhang , Polym. Chem. 2014, 5, 3298.

[9] S. Griggs , A. Marks , H. Bristow , I. McCulloch , J. Mater. Chem. C 2021, 9, 8099. 10.1039/d1tc02048j PMC8264852 34277009

[10] Z. Zhao , Z. Yin , H. Chen , L. Zheng , C. Zhu , L. Zhang , S. Tan , H. Wang , Y. Guo , Q. Tang , Y. Liu , Adv. Mater. 2017, 29, 1602410. 10.1002/adma.201602410 27922201

[11] a) J. T. E. Quinn , J. Zhu , X. Li , J. Wang , Y. Li , J. Mater. Chem. 2017, 5, 8654.

[12] J.‐S. M. Lee , A. I. Cooper , Chem. Rev. 2020, 120, 2171. 31990527 10.1021/acs.chemrev.9b00399 PMC7145355

[13] X. Wei , W. Zhang , G. Yu , Adv. Funct. Mater. 2021, 31, 2010979.

[14] H.‐J. Noh , S. Chung , M. S. Okyay , Y.‐K. Im , S.‐W. Kim , D.‐H. Kweon , J.‐P. Jeon , J.‐M. Seo , N.‐H. Kim , S.‐Y. Yu , Y. Reo , Y.‐Y. Noh , B. Kang , N. Park , J. Mahmood , K. Cho , J.‐B. Baek , Chem 2022, 8, 3130.

[15] M. Wang , M. Ballabio , M. Wang , H.‐H. Lin , B. P. Biswal , X. Han , S. Paasch , E. Brunner , P. Liu , M. Chen , M. Bonn , T. Heine , S. Zhou , E. Cánovas , R. Dong , X. Feng , J. Am. Chem. Soc. 2019, 141, 16810. 31557002 10.1021/jacs.9b07644

[16] a) W. Zhang , W. Lai , R. Cao , Chem. Rev. 2017, 117, 3717; 28222601 10.1021/acs.chemrev.6b00299

[17] M. Souto , D. F. Perepichka , J. Mater. Chem. C 2021, 9, 10668.

[18] E. Jin , M. Asada , Q. Xu , S. Dalapati , M. A. Addicoat , M. A. Brady , H. Xu , T. Nakamura , T. Heine , Q. Chen , D. Jiang , Science 2017, 357, 673. 28818940 10.1126/science.aan0202

[19] a) Y. Yang , C. Schäfer , K. Börjesson , Chem 2022, 8, 2217.

[20] L. Wang , B. Dong , R. Ge , F. Jiang , J. Xu , ACS Appl. Mater. Interfaces 2017, 9, 7108. 28192662 10.1021/acsami.6b14916

[21] K. S. W. Sing , D. H. Everett , R. A. W. Haul , L. Moscou , R. A. Pierotti , J. Rouquerol , T. Siemieniewska , Pure Appl. Chem. 1985, 57, 603.

[22] L. J. van der PAUW , Semiconductor Devices: Pioneering Papers, World Scientific, 1991, pp. 174–182.

[23] R. Lukose , M. Lisker , F. Akhtar , M. Fraschke , T. Grabolla , A. Mai , M. Lukosius , Sci. Rep. 2021, 11, 13111. 34162923 10.1038/s41598-021-92432-4 PMC8222355

[24] W. Choi , W. Kim , S. H. Hahn , K. Lim , H. Lee , M. Rhee , 2021 IEEE 23rd Electronics Packaging Technology Conference (EPTC), EPTC, Singapore 2021, pp. 23–27.

[25] U. Gösele , Q.‐Y. Tong , Annu. Rev. Mater. Sci. 1998, 28, 215.

[26] C. Cong , K. Li , X. Zhang , T. Yu , Sci. Rep. 2013, 3, 1195. 23378926 10.1038/srep01195 PMC3561624

