# Supplementary material for: A Rational Design of Isoindigo‐Based Conjugated Microporous n‐Type Semiconductors for High Electron Mobility and Conductivity
Source: Adv Sci (Weinh). 2023 Aug 17;10(29):2303562. doi: 10.1002/advs.202303562 (PMC10582460; doi:10.1002/advs.202303562)
Supplement: Supplementary file 1 — Supporting Information [file ADVS-10-2303562-s001.pdf]

ADVANCED SCIENCE
Open Access

Supporting Information

for *Adv. Sci.*, DOI 10.1002/advs.202303562

A Rational Design of Isoindigo-Based Conjugated Microporous *n*-Type Semiconductors for High Electron Mobility and Conductivity

Kayaramkodath Chandran Ranjeesh, Ayman Rezk, Jose Ignacio Martinez, Safa Gaber, Areej Merhi, Tina Skorjanc, Matjaž Finšgar, Gisha Elizabeth Luckachan, Ali Trabolsi, Bilal R. Kaafarani, Ammar Nayfeh* and Dinesh Shetty**

Supporting Information for

A Rational Design of Isoindigo-Based Conjugated Microporous n-type Semiconductors for High Electron Mobility and Conductivity

Kayaramkodath Chandran Ranjeesh,[a] Ayman Rezk,[b] Jose Ignacio Martinez,[c] Safa Gaber,[a] Areej Merhi,[d] Tina Skorjanc,[e] Matjaž Finšgar,[f] Gisha Elizabeth Luckachan,[a] Ali Trabolsi,[g] Bilal R. Kaafarani,*[d] Ammar Nayfeh,*[b] Dinesh Shetty* [a, h]

[a] Dr. K. C. Ranjeesh, S. Gaber, Dr. G. E. Luckachan, Prof. D. Shetty, Department of Chemistry, Khalifa University, PO Box 127788, Abu Dhabi, UAE.

E-mail: dinesh.shetty@ku.ac.ae.

[b] Dr. A. Rizk, Prof. A. Nayfeh, Department of Electrical Engineering and Computer Science, Khalifa University, PO Box 127788, Abu Dhabi, UAE. Email: ammar.nayfeh@ku.ac.ae.

[c] Dr. J. I. Martinez, Department of Low-dimensional Systems, Instituto de Ciencia de Materiales de Madrid-CSIC, C/ Sor Juana Inés de la Cruz 3, 28049 Madrid, Spain.

[d] A. Merhi, Prof. B. R. Kaafarani, Department of Chemistry, American University of Beirut, Beirut 1107-2020, Lebanon. Email: bilal.kaafarani@aub.edu.lb.

[e] Dr. T. Skorjanc, Materials Research Laboratory, University of Nova Gorica, Vipavska cesta 11c, 5270 Ajdovscina, Slovenia.

[f] Dr. M. Finšgar, Faculty of Chemistry and Chemical Engineering, University of Maribor, Smetanova ulica 17, 2000 Maribor, Slovenia.

[g] Prof. A. Trabolsi, Science Division, New York University Abu Dhabi, Saadiyat Island, PO Box 129188, Abu Dhabi, UAE; NYUAD Water Research Center, New York University Abu Dhabi (NYUAD), Saadiyat Island, PO Box 129188, Abu Dhabi, UAE.

[h] Prof. D. Shetty, Advanced Materials Chemistry Center (AMCC)
Khalifa University, P.O. Box 127788, Abu Dhabi, UAE

*Correspondence to: bilal.kaafarani@aub.edu.lb; ammar.nayfeh@ku.ac.ae.;
dinesh.shetty@ku.ac.ae

Content

1. Materials	Page 2
2. Synthesis	Page 2-5
3. Instruments and Methods	Page 5-12
4. Figures S1 to S17	Page 13-26
5. Table S1, S2	Page 22,27
7. References	Page 28-29

1. Materials

1,3,5-triethynylbenzene (Sigma-Aldrich), 1,3,6,8-tetraethynylpyrene (ET Co., Ltd.), 6-bromo-1H-indole-2,3-dione (Sigma-Aldrich), acetic acid (Merck), hydrochloric acid (Merck), triphenylphosphine (PPh_3) (TCI), chloroform, ($CHCl_3$) (Merck), methanol (MeOH) (Fisher Scientific), triethylamine (Et_3N) (Sigma-Aldrich), dimethylformamide (DMF) (Merck), tetrahydrofuran (THF) (Merck), $Pd(PPh_3)_4$ (Sigma-Aldrich), copper iodide (CuI) (Sigma-Aldrich). The (E)-6,6'-dibromo-1,1'-dihexyl-[3,3'-biindolinylidene]-2,2'-dione monomers used in this study were obtained using our reported procedures. [1] All the polymerization reactions were carried out in oven-dried 100 ml Schlenk flask under an air atmosphere unless otherwise mentioned.

2. Synthesis

Synthesis of 6,6'-dibromo-[3,3'-biindolinylidene]-2,2'-dione

The title compound was synthesized according to a modified literature procedure. [1] A mixture of 6-bromoindoline-2,3-dione (1 g, 4.42 mmol), 6-bromoindolin-2-one (1 g, 4.71 mmol) in acetic acid (30 mL) and hydrochloric acid (0.2 mL, 38%) was refluxed overnight. The brick red reaction mixture was left to cool at room temperature and then filtrated by suction filtration. The resulting crude product was recrystallized by 1,2-dichlorobenzene to afford a dark brown solid (1.83 g, 98%), m.p. > 260 °C. ^{1}H-NMR (500 MHz, DMSO-d_6): δ 11.11 (s, 2H), 9.00 (d, J = 8.5 Hz, 2H), 7.19 (d, J = 8.5 Hz, 2H), 6.99 (d, J = 2 Hz, 2H) ppm. ^{13}C-NMR (125 MHz, DMSO-d_6): δ 169.35, 146.01, 133.15, 131.39, 126.20, 124.47, 121.23, 112.87 ppm.

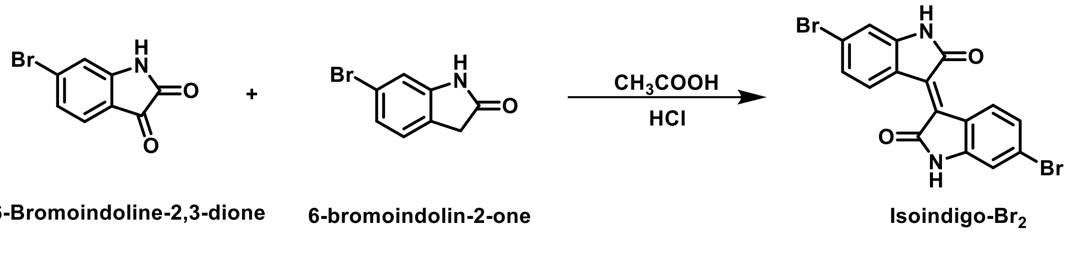

Synthesis of (*E*)-6,6'-dibromo-1,1'-dihexyl-[3,3'-biindolinylidene]-2,2'-dione:

The title compound was synthesized according to a modified literature procedure.[1] In a Schlenk tube, under argon, a solution of (*E*)-6,6'-dibromo-[3,3'-biindolinylidene]-2,2'-dione (1 g, 2.38 mmol) and anhydrous potassium carbonate (2 g, 14.47 mmol) was prepared in DMF (50 mL). Under argon, 1-bromohexane (2 mL, 14.30 mmol) was added, and the

reaction mixture was heated at 110 °C for 72 hrs. After cooling to room temperature, the reaction was quenched with distilled water (180 mL) and filtrated. The crude product was purified by silica gel column chromatography (*n*-hexane: dichloromethane; 3:2) as mobile phase. The obtained red solid was recrystallized from toluene-hexane to afford a red solid (1.1 g, 78%), m.p. 170-172 °C. ^{1}H-NMR (500 MHz, CDCl3): δ 9.06 (d, J = 8.5 Hz, 2H), 7.15 (dd, J_1 = 8.5 Hz, J_2 = 2 Hz, 2H), 6.91 (d, J = 2 Hz, 2H), 3.71 (t, J = 7.5 Hz, 4H), 1.65 (p, J = 7.5 Hz, 4H), 1.30 (m, overlapping, 12 H), 0.87 (t, J = 7 Hz, 6H) ppm. ^{13}C NMR (125 MHz, $CDCl_3$): δ 167.74, 145.77, 132.67, 131.17, 126.74, 125.14, 120.40, 111.32, 40.27, 31.45, 27.36, 26.66, 22.55, 14.03.

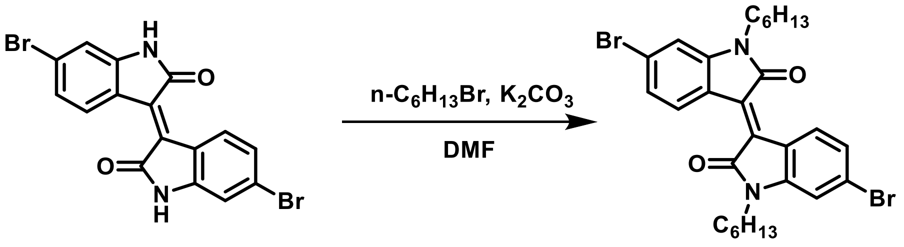

Isoindigo-Br_2 1

Synthetic procedure for BI:

1,3,5-triethynylbenzene (150 mg, 0.99 mmol) and (E)-6,6'-dibromo-1,1'-dihexyl-[3,3'-biindolinylidene]-2,2'-dione (881.5 mg, 1.50 mmol) were charged in a dried Schlenk flask (100 mL). The mixture of DMF and Et_3N (1:1 v/v) (20: 20 mL) was added as a solvent to the Schlenk tube under an inert atmosphere. The mixture was degassed and purged with argon or N_2 for 30 minutes. Tetrakis (triphenylphosphine) palladium(0) (Pd $(PPh_3)_4$) (230.84 mg, 0.2 mmol), copper(I) iodide (CuI) (38 mg, 0.2 mmol), and triphenylphosphine (PPh_3) (52 mg, 0.2 mmol) were introduced and purging was continued for another 10 minutes. The resulting solution was sonicated for 5 minutes and stirred at 120 °C for 48 hours. The mixture was then cooled down to room temperature, and the precipitated polymer was centrifuged and washed with methanol, water, chloroform, and acetone, respectively. Further purification of the polymer was carried out by Soxhlet extraction with methanol for 48 h. The product was dried under vacuum for 24 h at 70 °C and obtained as a dark reddish-brown powder (yield: 83%, Scheme S1).

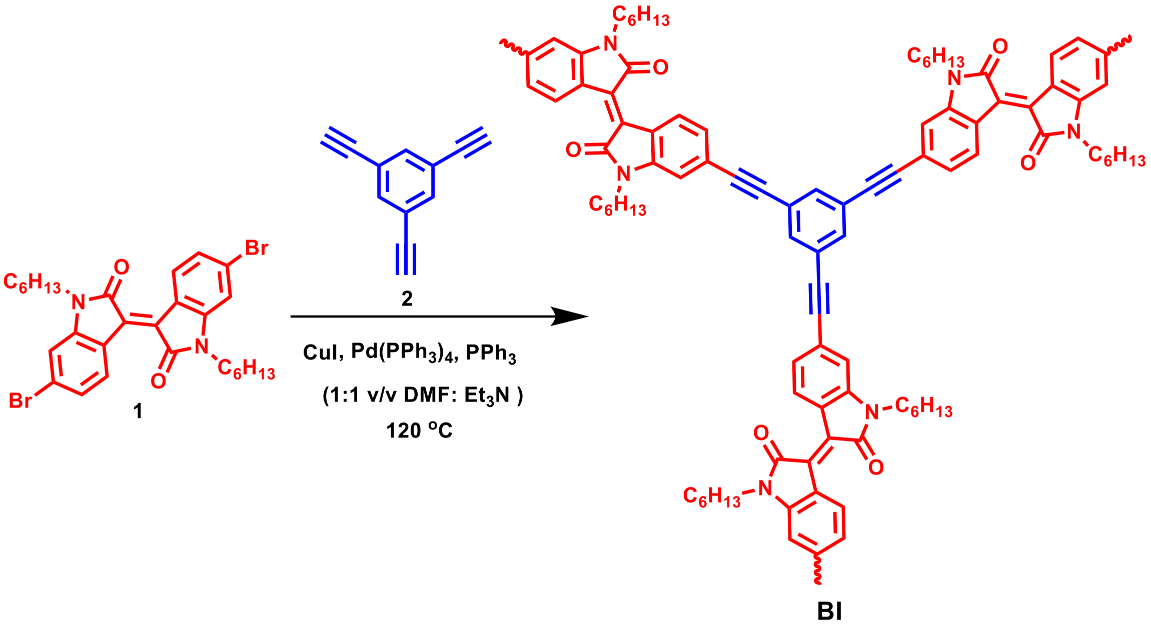

Scheme S1. Synthetic scheme of **BI**.

Synthetic procedure for PI:

1,3,6,8-Tetraethynylpyrene (150 mg, 0.50 mmol) and (E)-6,6'-dibromo-1,1'-dihexyl-[3,3'-biindolinylidene]-2,2'-dione (591.6 mg, 1.01 mmol) were charged in a dried Schlenk flask (100 mL). The mixture of DMF and Et_3N (1:1 v/v) (15: 15 mL) was added as a solvent to the Schlenk tube under an inert atmosphere. The mixture was degassed and purged with argon or N_2 for 30 minutes. Tetrakis (triphenylphosphine) palladium(0) (Pd $(PPh_3)_4$) (116.2 mg, 0.10 mmol), copper(I) iodide (CuI) (19 mg, 0.10 mmol), and triphenylphosphine (PPh_3) (26 mg, 0.10 mmol) were introduced and purging was continued for another 10 minutes. The resulting solution was sonicated for 5 minutes and stirred at 120 °C for 48 hours. The mixture was then cooled down to room temperature, and the precipitated polymer was centrifuged and washed with methanol, water, chloroform, and acetone, respectively. Further purification of the polymer was carried out by Soxhlet extraction with methanol for 48 h. The product was dried under vacuum for 24 h at 70 °C and obtained as a dark reddish-brown powder. (Yield: 85 %, Scheme S2).

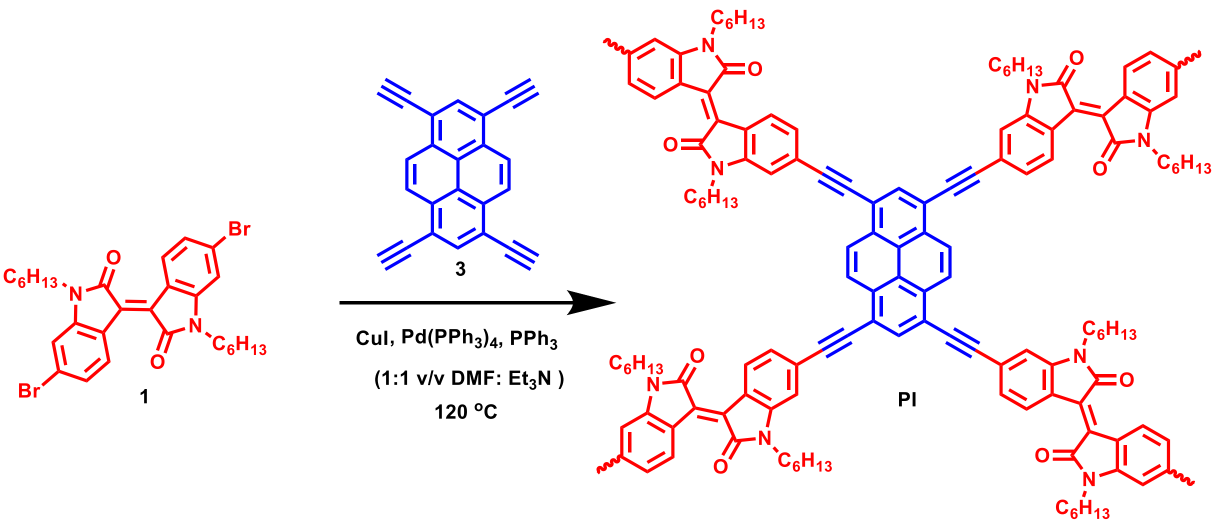

Scheme S2. Synthetic scheme of **PI**.

3. Instruments and Methods

Powder X-ray diffraction (PXRD): Powder X-ray diffraction measurements were performed on Rigaku Smart Lab II with Cu K_α (λ = 1.5405 Å) radiation source operating at 40 kV and 40 mA. The patterns were recorded with a divergent slit of 1/16° over the 2θ range of 2–50° with step size = 0.02°.

Fourier transform infrared (FT-IR): FT-IR spectra were taken on a Bruker Optics ALPHA-E spectrometer with a universal Zn-Se ATR (attenuated total reflection) accessory in the 600- 4000 cm^{-1} region or using a Diamond ATR (Golden Gate) with 24 scan rate and 4 cm^{-1} resolution.

Solid-state ^{13}Carbon Cross-Polarization Magic Angle Spinning (CP MAS): ^{13}C CP MAS NMR spectra of the COFs were recorded on a Bruker Avance NEO 500MHz NMR spectrometer using a 4.0 mm MAS probe at ambient temperature and a magic angle spinning rate of 12.0 kHz. Spectra were acquired using a CP contact time of 2000us, a recycle delay of 2 sec and a total number of 42200 scans. ^{13}C chemical shifts were externally referenced to the adamantane CH_2 signal at 38.46 ppm. NMR data were processed using the software "TopSpin 4.1.4".

N_2 adsorption

Porosity analyses were performed on the Anton Paar Autosorb iQ combined physisorption and chemisorption instrument. For each measurement, 20–30 mg of COF samples were

used. The samples were activated at 80 °C for 16 hours before being subject to N_2 gas adsorption in liquid N_2 bath (77 K) to collect full isotherms. Surface areas were calculated using the multipoint Brunauer – Emmett – Teller (BET) model, and pore size distributions were found using the non-local density functional theory (NLDFT). P and P_0 are the equilibrium at the saturation pressure of N_2.

Scanning Electron Microscopy (SEM): The FEI Nova NanoSEM 650 was employed for the SEM analysis of COFs samples. It combines an electron column with semi-in-lens detectors and an in-the-lens Schottky field emission gun to deliver ultrahigh-resolution with a wide range of probe currents (1 pA to more than 200 nA). With a voltage of 2.0 keV, the SEM images were recorded. The SEM samples were prepared by drop-casting (10 μL) of COFs dispersions in isopropyl alcohol on a silicon substrate and drying in air. After complete evaporation of the solvent, the samples were coated with Pt (nano-sized film) using the JEOL JEC-300FC Auto Fine before SEM analysis.

Transmission Electron Microscopy (TEM): The TEM images were recorded by FEI Tecnai TEM 20 kV. The TEM samples were prepared by the COFs powder dispersed in isopropyl alcohol solvent by 15-minute sonication, drop cast on carbon-coated copper grids TEM Window (TED PELLA, INC. 300 mesh), and allowed to dry overnight in desiccators.

X-ray photoelectron spectroscopy (XPS)

XPS measurements were performed using a Supra+ instrument (Kratos, Manchester, UK) equipped with an Al K_α excitation source and a monochromator. The charge neutralizer was on during the measurements. The take-off angle was 90°. XPS measurements and data processing were performed using ESCApe 1.5 software (Kratos). The powder samples were placed on a carbon tape attached to the silicon wafer. The area analysed was 300 by 700 microns. The measurements were performed at a pass energy of 20 eV. The base pressure in the main analysis chamber was $8 \cdot 10^{-8}$ mbar. The binding energy scale was corrected based on the C-C/C-H peak at 284.8 eV in the C 1s spectrum.

Cyclic Voltammetry (CV)

The electrochemical cyclic voltammetry experiment was carried out on a CHI 600 electrochemical workstation using a platinum disk, a platinum wire, and an Ag/Ag^+ electrode as working, auxiliary, and reference electrodes, respectively. The experiment was

carried out in a deoxygenated anhydrous acetonitrile solution of tetra-n-butylammonium hexafluorophosphate (0.1 M) under an atmosphere of nitrogen at a scan rate of 50 mV s^{-1}. The potential of the Ag/Ag^{+} reference electrode was internally calibrated using the known energy level (-4.8) of the ferrocene/ferrocenium redox pair (Fc/Fc^{+}). The HOMO and LUMO energy levels were deduced from the oxidation onset (E^{ox}onset) and the reduction onset (E^{red}onset), respectively, using the following equations: E_{HOMO} = -(4.8+E^{ox}onset) and E_{LUMO}= -(4.8+E^{red}onset).

Ultraviolet photoelectron spectroscopy (UPS)

UPS analyses were performed on a Thermo Fisher Scientific Instruments UK, Sr.No.-KAS2020. Ultraviolet photoemission valance band spectrum (UPS) was performed on Thermo ESCALAB Xi+ with Helium light source (h*ν*=21.2 eV). Photoelectron spectrometer was under an ultrahigh vacuum of about 3×10^{-9} Torr at 300 K. For UPS measurements, the samples were biased at –10 V for **BI** and -12 V for **PI** to observe the low-energy secondary electron cutoff. The samples were prepared by drop-casting **BI** and **PI** dispersions in isopropanol (10 μL) on a silicon substrate (freshly cleaned with pure isopropanol; dried) and dried under Ar in a desiccator. After complete evaporation of the solvent, the sample was directly used for UPS analysis.

Method for chemical exfoliation and thin preparation of CMPs (**BI** and **PI**)

The solution-based thin films, used in the Hall effect measurements, are developed by chemical exfoliation. 50 mg of the CMP (**BI** and **PI**) powder is dispersed in 50 mL NMP (N-Methyl-2-Pyrrolidone) followed by sonicating the mixture for 8 hours using an ultra-probe-sonicator at a 10s/2s duty cycle in an ice bath. The solution is then centrifuged at 1500 rpm for 60 min to filter the unexfoliated particles and at 7500 rpm for 30 min to remove the insoluble impurities. After filtering, the CMPs are re-dispersed in 50 mL of IPA.

Hall effect measurement (HMS)

HMS was employed to characterize conductivity type, mobility, and carrier density in **BI** and **PI** thin films using Ecopia HMS-5000. The electrical contact was made by placing

silver conductive paste on the four corners of thin films. The sample then was measured at a constant current in dark conditions at 25 ºC and a typical magnetic field of 0.55 T.

Surface Plasma Treatment

To employ plasma treatment on the surface of drop-casted thin films. Radio frequency (RF) glow discharge in the ambient gas of oxygen was used. By altering the surface of the substrate using plasma, extra charged electrons are lingering on the surface of the thin film, seeking lower energy states. The surface oxygen plasma activation is optimized in a RIE plasma reactor (SAMCO's RIE-200iP) with 90 W RF power at 50 sccm flow rate for 3 min.

Detailed information on Hall effect measurements and calculations

The substrate was chosen as a p-doped (100) silicon (n-Si) wafer, with 1-10 Ω.cm resistivity, and topped with a 300 nm wet thermal oxide. Before drop casting, 1×1 cm^2 square pieces of this wafer were first cleaned in acetone using ultrasonication and then rinsed with IPA, deionized water, and dried with N_2 spray. 20 µL of exfoliated polymer were drop-casted on the substrates for a total thickness of around 0.2 µm. The silver paste was applied at the corners of the films, which served as contacts. Then the measurements were carried out using the Ecopia HMS-5000 Hall effect measurement system by applying a constant current (1 mA) in dark conditions at 25 ºC and a typical magnetic field of 0.55 T.

The Hall-Effect measurement relies on the application of a magnetic field perpendicular to the direction of current flow in a conducting sample, resulting in a transverse voltage known as the Hall voltage. This voltage can be measured to extract information about the polymer material's electronic properties. Here's a summary of the process flow used to conduct the Hall-effect measurements:

1. **Sample preparation:** A 1×1 cm^2 square piece of the sample is prepared for measurements. The silver paste is applied at the corners of the sample, which served as current contacts, while a magnetic field is applied perpendicular to the current path.
2. **Current and magnetic field setup:** A constant current, I of 1 mA, is passed through the sample using the contacts. Simultaneously, a magnetic field, B of 0.55 T, is applied perpendicular to the current direction. The magnetic field is

sufficiently strong to cause a measurable Hall voltage but not strong enough to affect the polymer's electronic properties.

3. **Hall voltage measurement:** As the current flows through the sample under the influence of the magnetic field, a voltage perpendicular to both the current and the magnetic field, known as the Hall voltage (V_H), is generated. The Hall voltage is measured using a voltmeter.
4. **Hall coefficient calculation:** The Hall coefficient (R_H) is related to carrier density (n) and mobility (μ). It can be calculated using the following formula:

$$R_H = \frac{V_H}{B \times I} t \qquad (1)$$

Where t is the polymer film thickness in meters. Hall coefficient (R_H) represents the ratio of the Hall voltage (V_H) to the product of the magnetic field strength (B) and current (I).

5. **Carrier density calculation:** The Hall coefficient (R_H) allows the calculation of the carrier density (n) through the equation:

$$n = \frac{1}{R_H \times q} \qquad (2)$$

where q is the elementary charge.

6. **Mobility calculation:** The carrier mobility (μ) can be calculated using the formula:

$$\mu = \frac{R_H}{\rho} \qquad (3)$$

where ρ is the resistivity.

Theoretical Framework

In order to shed some light from the theoretical perspective into the transport properties of both **PI** and **BI** compounds we have computed, as a proof of concept, the intrinsic carrier mobilities for the two monolayers within the deformation potential (DP) formalism.[2] to describe charge transport in nonpolar semiconductors. DP approach can be simplified into an effective mass approximation. [3,4] Given the structural symmetry of both systems, we consider the elastic modulus and effective mass purely isotropic within the 2D layered structures. Thus, the main DP parameters to be investigated are the elastic constant, the

deformation constant and the effective mass.[5] The equation used to estimate the 2D sheet intrinsic carrier mobility is the following:

$$\mu_{2D} = \frac{e\tau}{m^*} = \frac{e\hbar^3 C_{2D}}{k_B T |m^*|^2 {E_1}^2}, \qquad \text{(eq. 1)}$$

where m^* is the effective mass; τ is the relaxation time, T is the temperature, E_1 is the DP constant, which represents the strain-induced shift of the band edges, and C_{2D} is the elastic modulus, obtained from the lattice distortion by the strain. The main assumption is our case is the isotropy in the semiconductor 2D sheets, where electrons and phonons will behave in the same way in the two lattice directions.

Computational details. We have carried out a large battery of Density Functional Theory (DFT) based calculations to investigate the structural and electronic properties of both compounds, as well as all the DP parameters to get the intrinsic carrier mobilities. As a starting point, molecular fragments involved in the formation of the systems were studied within the framework of the DFT implemented within the Gaussian16 program.[6] For this purpose, we used as level of theory the hybrid generalized gradient approximation (GGA) long-range corrected hybrid functional CAM-B3LYP [7] together with the cc-pVDZ [8] basis set. All geometrical freedom degrees were allowed to vary independently. The calculated geometries of the molecular building blocks were confirmed as minima by frequency calculations. Periodic boundary conditions were used to perform simultaneous structure + cell optimizations of different stacked 3D layered system models based on their canonical 2D layer structures. The optimized molecular building-blocks were used to construct the different 2D networks with the QUANTUM EXPRESSO plane-wave DFT code [9] by using the GGA-PBE functional [10] to account for the exchange-correlation (XC) effects, and the Grimme DFT-D3 semi-empirical efficient vdW correction to include dispersion forces and energies in conventional DFT functionals [11] Ultra-soft pseudopotentials were used to model the ion-electron interaction within the atomic species.[12] Brillouin zones were sampled by means of optimal [2×2×1] and [2×2×8] Monkhorst-Pack grids[13] for the 2D layers and 3D crystals, respectively. One-electron wave-functions are expanded in a basis of plane-waves with a kinetic energy cutoff of 40 and 300 Ry for the kinetic energy

and electronic density, respectively, which achieve sufficient accuracy to guarantee a full convergence in total energy and electronic density. In the full structure + cell optimizations for the different 3D crystal models the atomic relaxations were carried out within a conjugate gradient minimization scheme until the maximum force acting on any atom was below 0.02 eV $Å^{-1}$, including relaxation of interlayer distances. The crystal-bulk models were analyzed for their both eclipsed (AA) and staggered (AB) configurations, and some other intermediate ones.

Computation of the DP parameters. Within the DP theory we only consider the acoustic-phonon scattering mechanism, and the fundamental DP constants to be computed are the elastic constant (C_{2D}), the deformation constant (E_1) and the effective electron mass (m_e^*) for both **PI** and **BI** compounds. $C_{2D} = [\partial^2 E/ \partial\delta^2]/\Delta$, where E is the total energy of the unit cell, δ is the uniaxial strain applied to both lattice directions simultaneously, and $\Delta = S/S_0$ describes the change in the surface area at a dilatation. C_{2D} is then obtained by parabola fitting energy-strain curves. By dilating the lattice in the same way as for the calculation of C_{2D}, E_1can be calculated as $dE/d\delta$, where E is the energy of CB edge for the δ strain. The effective mass, m^*, is calculated using $\hbar^2[\partial^2\varepsilon(k)/ \partial k^2]^{-1}$, where ε(k) is the band energy, in this case, at Γ point to mimic the average electronic properties obtained from the experiments. For parabolic bands, the electron will move much like a free particle with m*, which results in constant effective masses.

After the simultaneous cell + structure relaxations, and after testing a bunch of different interlayer stacking configurations, the results of the simulations yield a structure for the **PI** compound with a unit cell of [2.28×2.28] nm^2 and an angle between lattice parameters of 104.1°, with a preferential stacking configuration close to the eclipsed (AA) one and an interlayer distance of 5.11 Å, whilst the **BI** compound exhibits a perfectly hexagonal P6 symmetry with a unit cell of [3.55×3.55] nm^2 and an angle between lattice parameters of 60°. In this case, the preferential stacking fashion is a perfectly eclipsed AA configuration with an interlayer distance of 4.98 Å. Regarding their electronic properties, in both cases, they exhibit a clear narrow-gap semiconducting character with band-gaps at X- and Γ-points of 1.06 and 1.57 eV for the **PI** and **BI** compounds, respectively, in good agreement

with the experimental evidence. Based on the DFT calculations, the acoustic-phonon-limited electron mobilities at room temperature (300 K) were obtained with the values E_1, C_{2D}, and m_e^*. We have obtained values for C_{2D} of 145.2 and 223.5 N m^{-1} for **PI** and **BI**, respectively; values for E_1 of -5.7 and -6.5 eV for electrons for **PI** and **BI**, respectively; and, finally, values of m_e^* of 1.42 and 3.95 m_e for **PI** and **BI**, respectively. All these values, according to (eq. 1), provide intrinsic electron mobilities of 44.7 and 8.7 cm^2 V^{-1} s^{-1}. Considering the use of only the acoustic phonon scattering mechanism approximation, together with the fact that the theoretical systems are highly idealized, the comparison between the experimental and theoretical mobilities is fairly good.

4. Figures

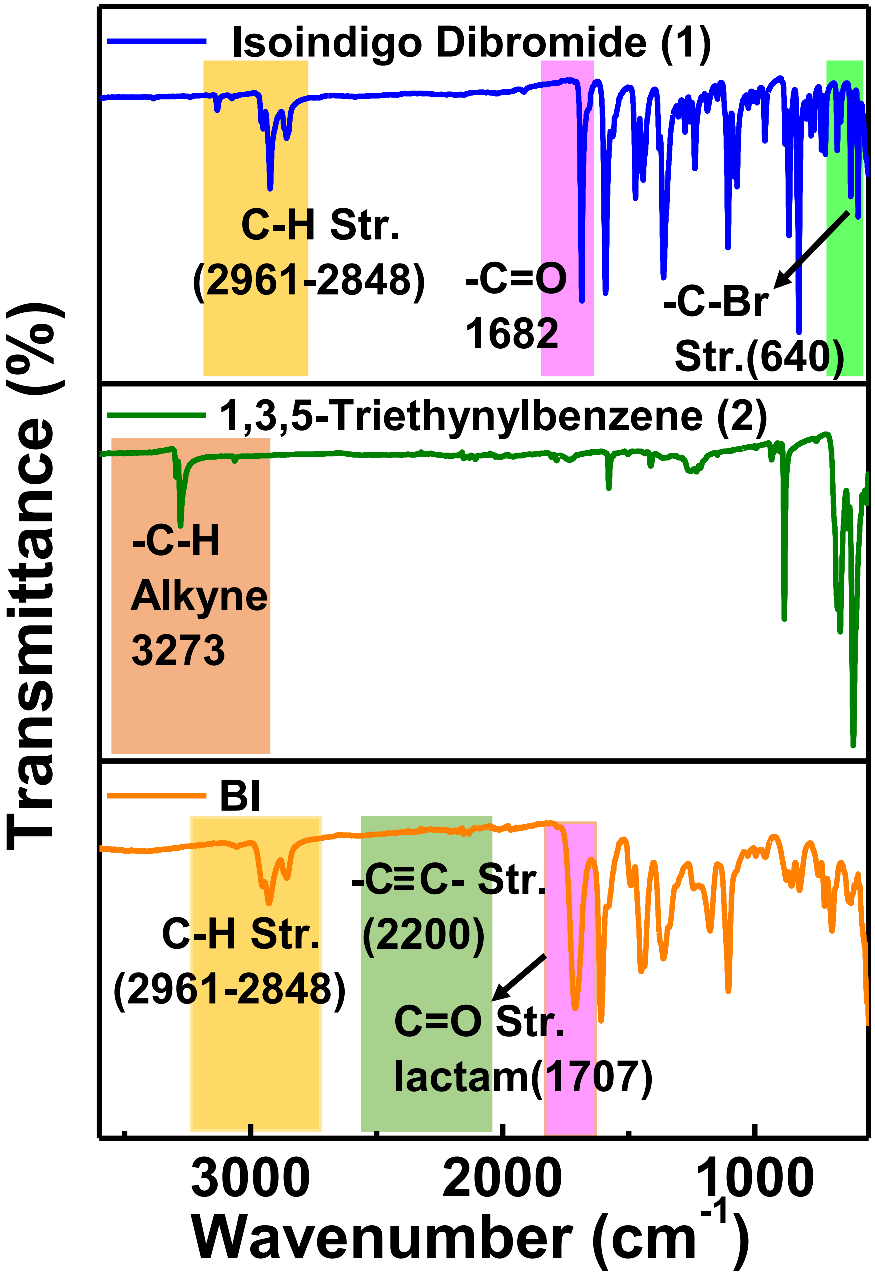

Figure S1. FT-IR spectra of **BI** and its constituent monomers.

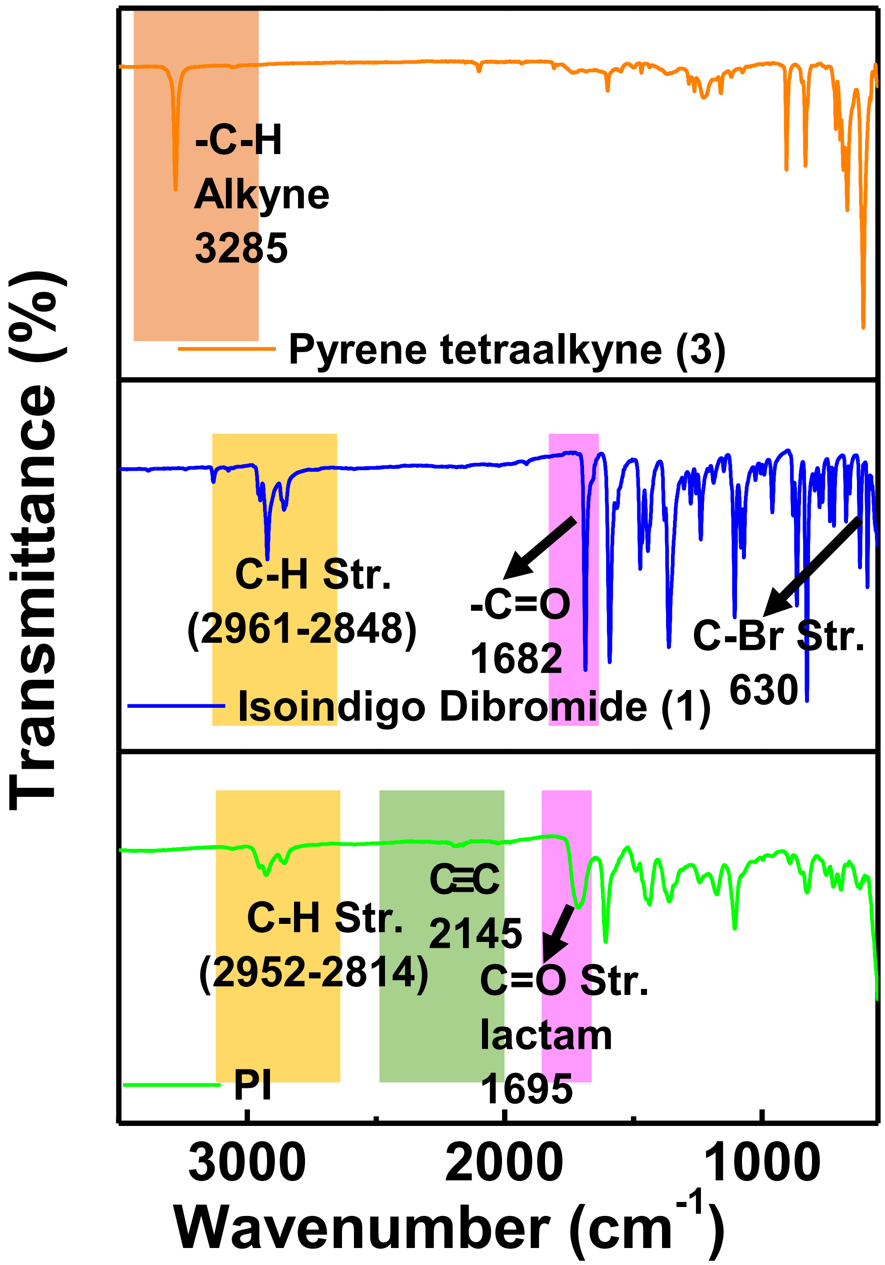

Figure S2. FT-IR spectra of **PI** and its constituent monomers.

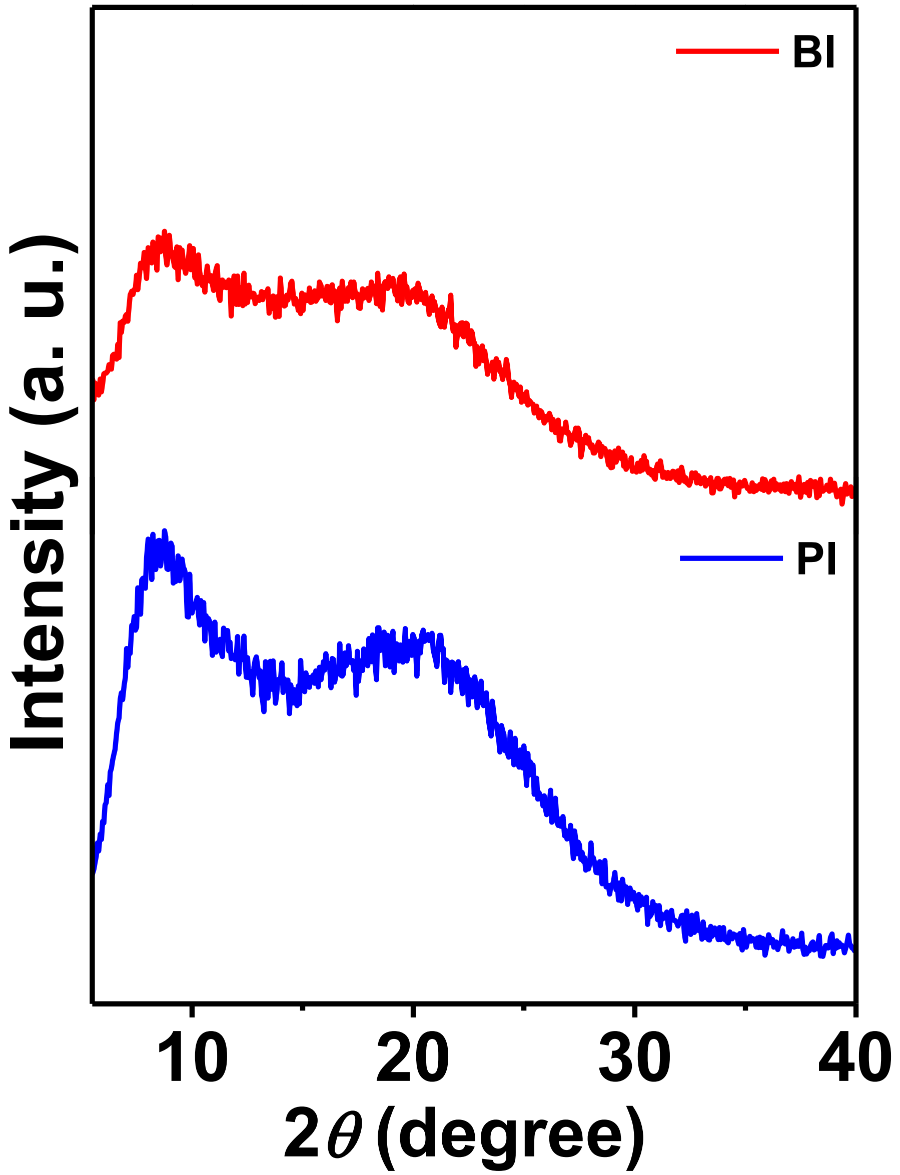

Figure S3. The PXRD patterns of **BI** and **PI**.

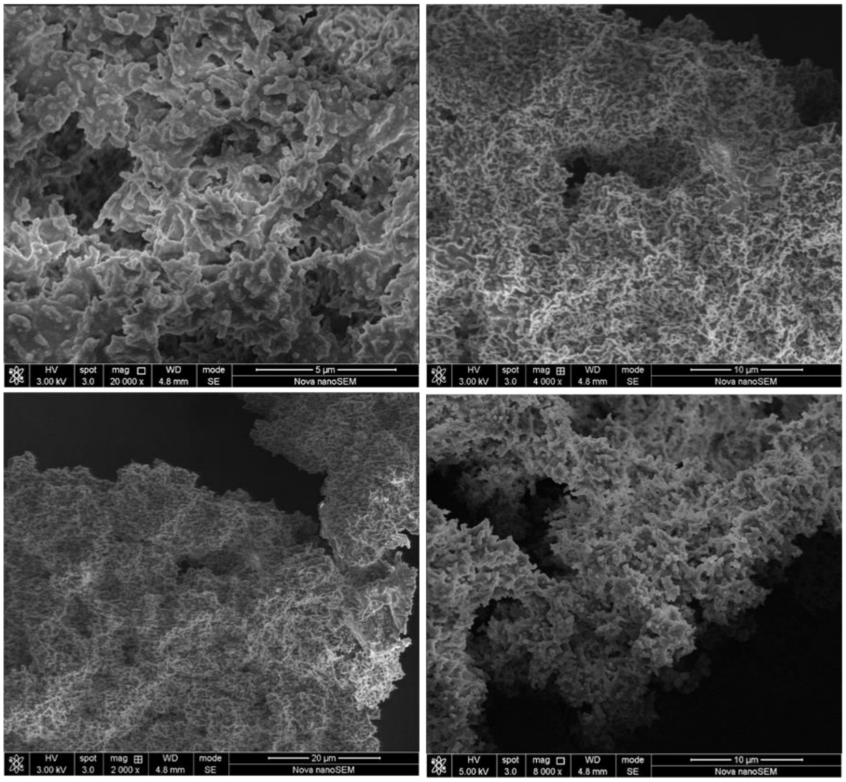

Figure S4. SEM images of **BI**.

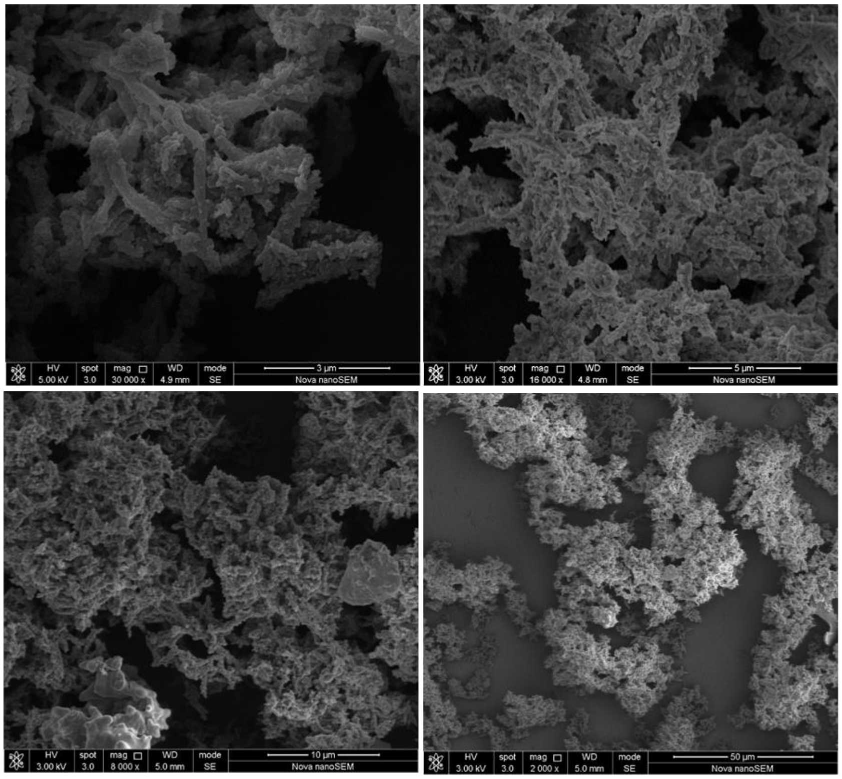

Figure S5. SEM images of **PI**.

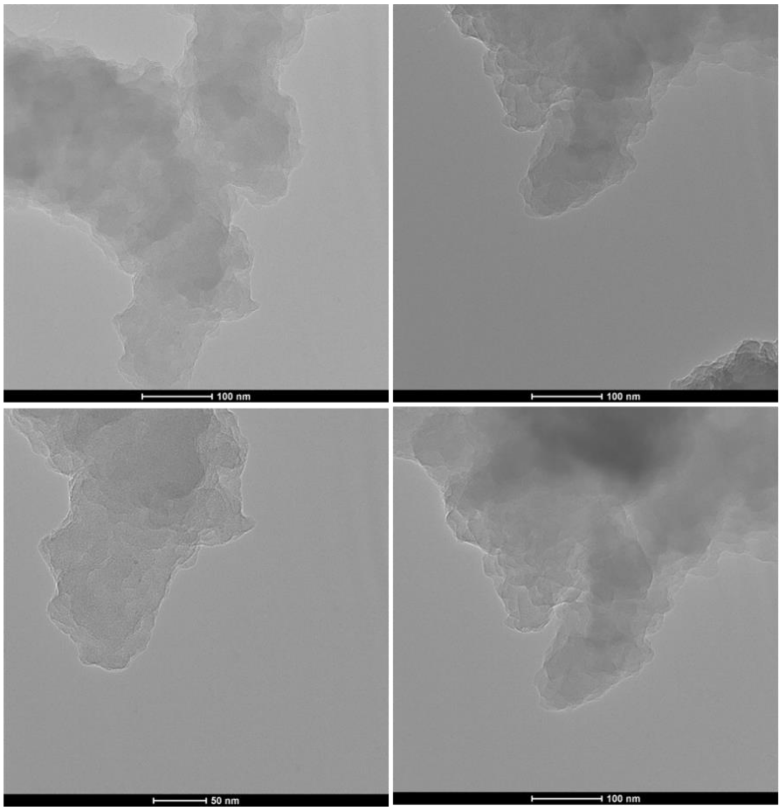

Figure S6: TEM images of **BI**.

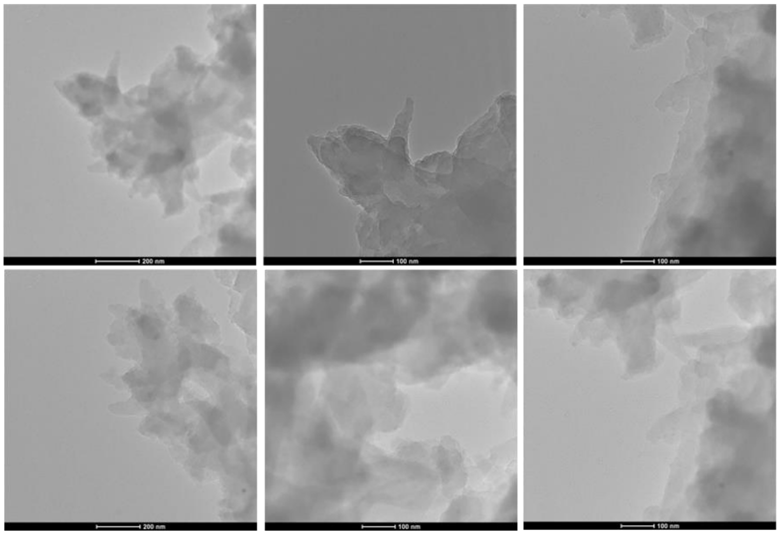

Figure S7: TEM images of **PI**.

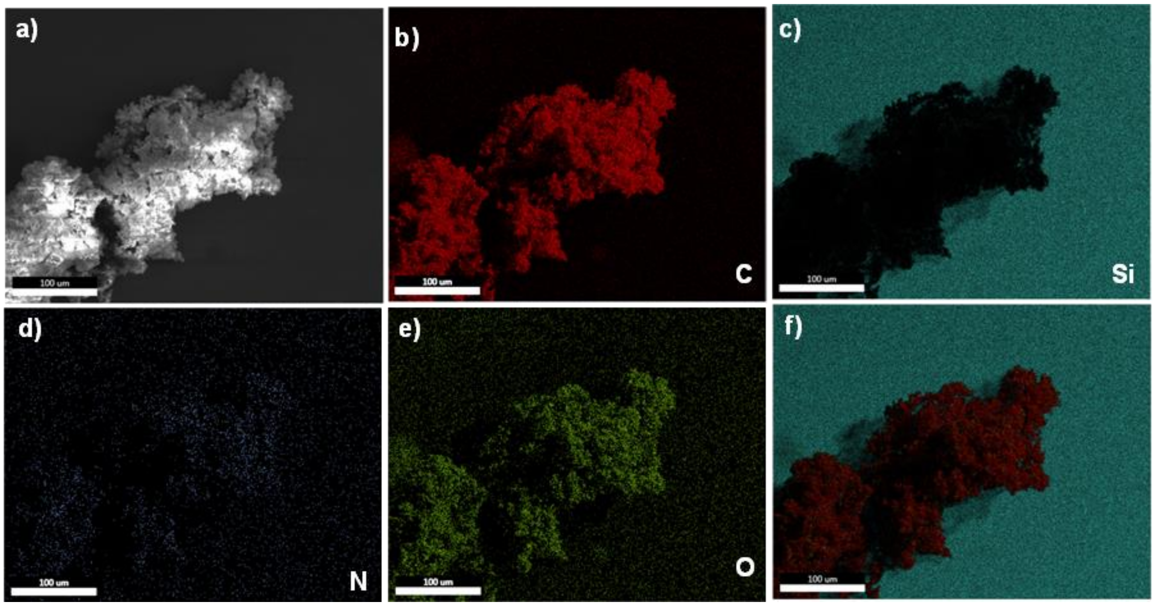

Figure S8: SEM-EDX elemental mapping of **BI**. a) Reference image of mapped material, b) carbon (C), c) silicon background (Si), d) nitrogen (N), e) oxygen (O) f) elemental overlay of **BI**.

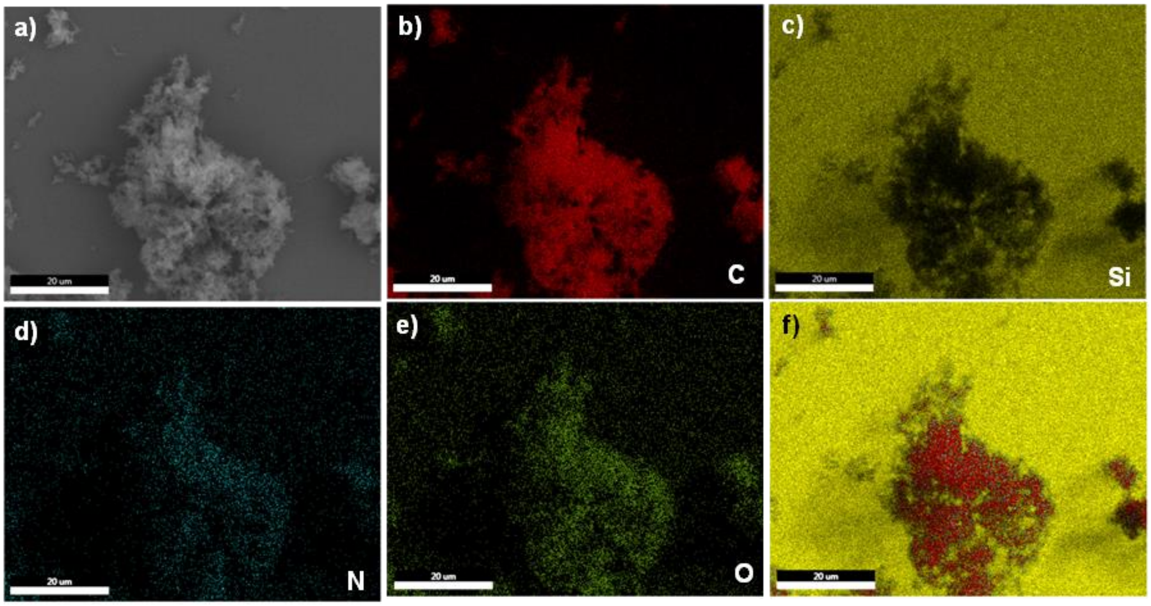

Figure S9: SEM-EDX elemental mapping of **PI**. a) Reference image of mapped material, b) carbon (C), c) silicon background (Si), d) nitrogen (N), e) oxygen (O) f) elemental overlay of **PI**.

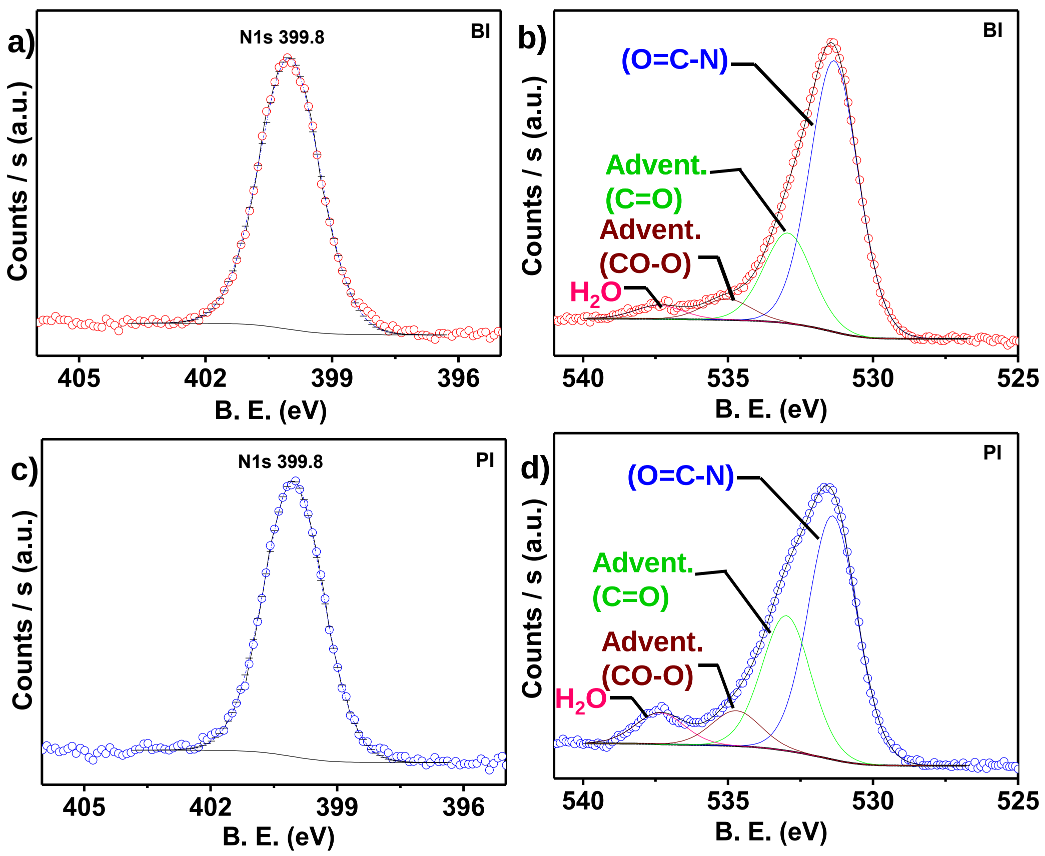

Figure S10. XPS spectra for a) N 1s b) O 1s of **BI**, and c) N 1s d) O 1s of **PI**.

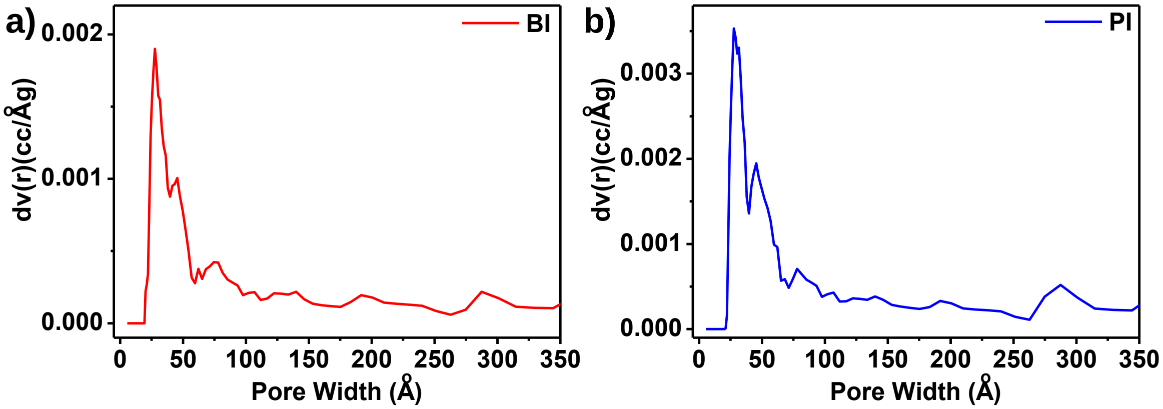

Figure S11. Pore-size distribution profile of a) **BI** and b) **PI**.

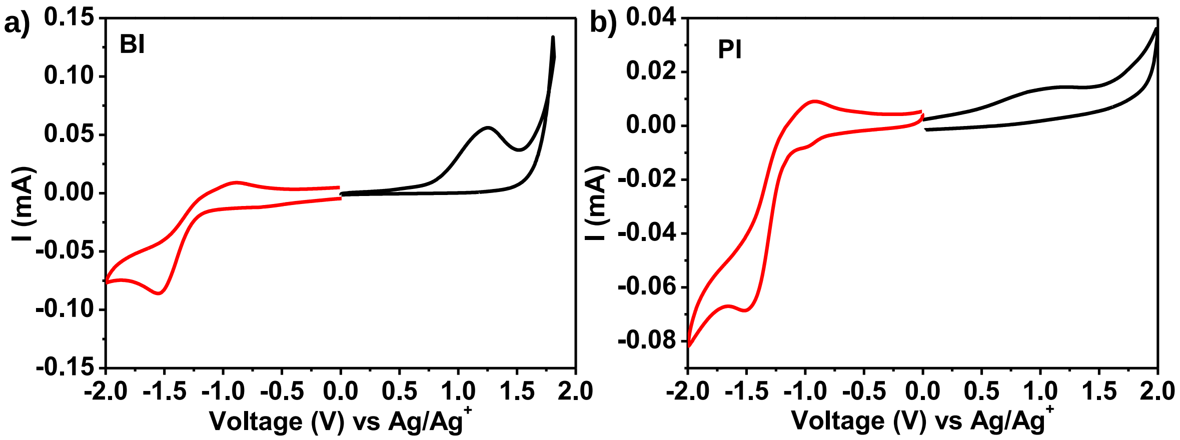

Figure S12. Cyclic voltammograms of a) **BI** and b) **PI** at a scan rate of 50 mV s^{-1}

Table S1. Electrochemical properties of **BI** and **PI**.

**Sample Code**	E^{ox}_{onset}	E^{red}_{onset}	E_{HOMO}	E_{LUMO}	Eg
**BI**	0.81 V	-1.221 V	- 5.61 eV	- 3.579 eV	2.031 eV
**PI**	0.426 V	-1.150 V	- 5.226 eV	-3.627 eV	1.599 eV

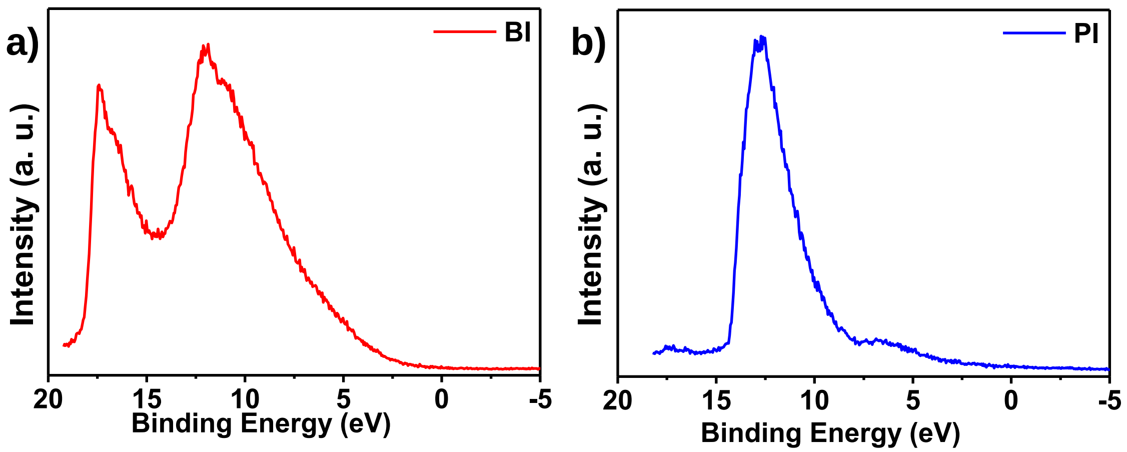

Figure S13. UPS spectrum of a) **BI** at -10 V bias Scan + 3 eV and b) **PI** at -10 V bias Scan + 2 eV. The work function **(**ϕ) was derived by subtracting the high-binding energy cut-off (16.4158 eV for **BI** 16.5412 eV for **PI)**; **BI(**ϕ) = 21.22 eV - 16.4158 eV= 4.805 eV, **PI(**ϕ) =21.22 eV- 16.5412 = 4.6788 eV from the radiation energy (21.22 eV; from non-voltage bias condition) Hence E_f for **BI** = -4.805 eV vs vacuum and E_f for **PI** = -4.805 eV vs vacuum. The low-energy tail of the UPS spectrum was used to determine the position of

the VBM as 1.037 eV for **BI** and 1.14 eV for **PI** below the Fermi level. As a result, the VBM was calculated to be for **BI=** - 4.805 eV-1.037= ($E_{HOMO/\ VBM}$) eV= -5.842 eV; VBM for **PI =** -4.6788-1.14= -5.818 eV ($E_{HOMO/\ VBM}$). Using the calculated optical bandgap of **BI=** 1.76 eV and **PI =** 1.58 (Fig. 3), the calculated conduction band minimum (CBM) was found to be -4.082 eV vs vacuum ($E_{CBM/\ LUMO}$= $E_{HOMO/\ VBM+}$ $Eg^{\ opt.}$ = -5.842 eV+ 1.76 eV) for **BI** whereas -4.258 eV vs vacuum ($E_{CBM/\ LUMO}$= $E_{HOMO/\ VBM+}$ $Eg^{\ opt.}$ = -5.818 eV+1.58 eV) for **PI**. This implies that **BI** and **PI** Fermi level is near the conduction band and therefore materials are n-type.

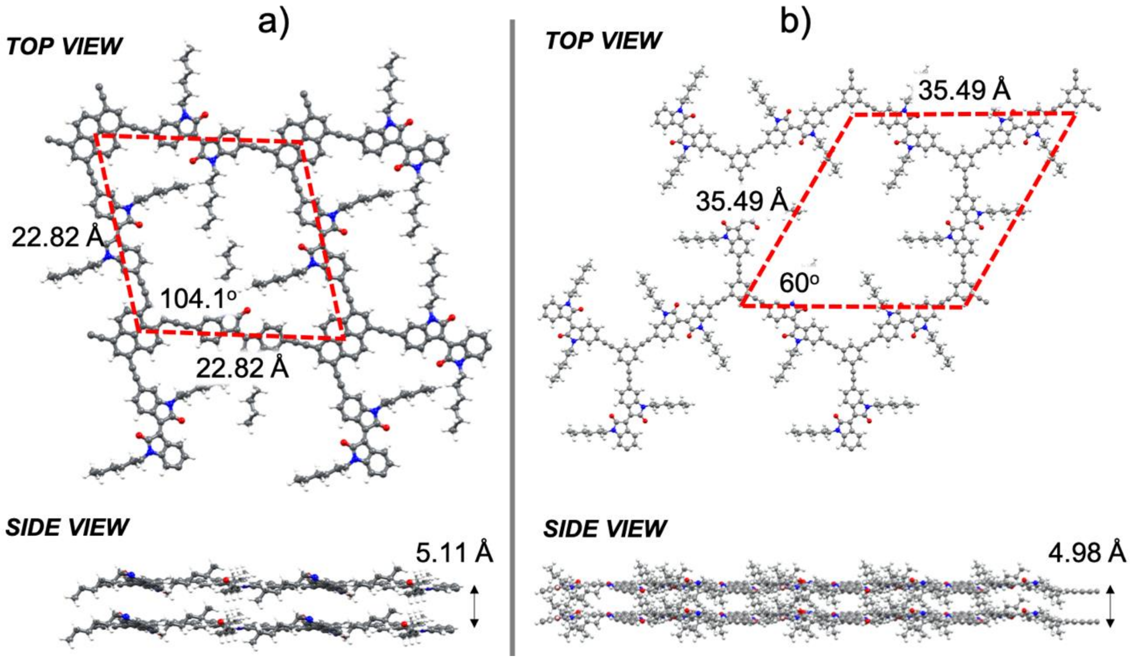

Figure S14. Top and side pictorial views of the most stable DFT optimized structures for the a) **PI** and b) **BI** compounds, indicating in both cases the resulting optimized unit cell and interlayer distance. White, grey, blue and red spheres represent H, C, N and O atoms, respectively.

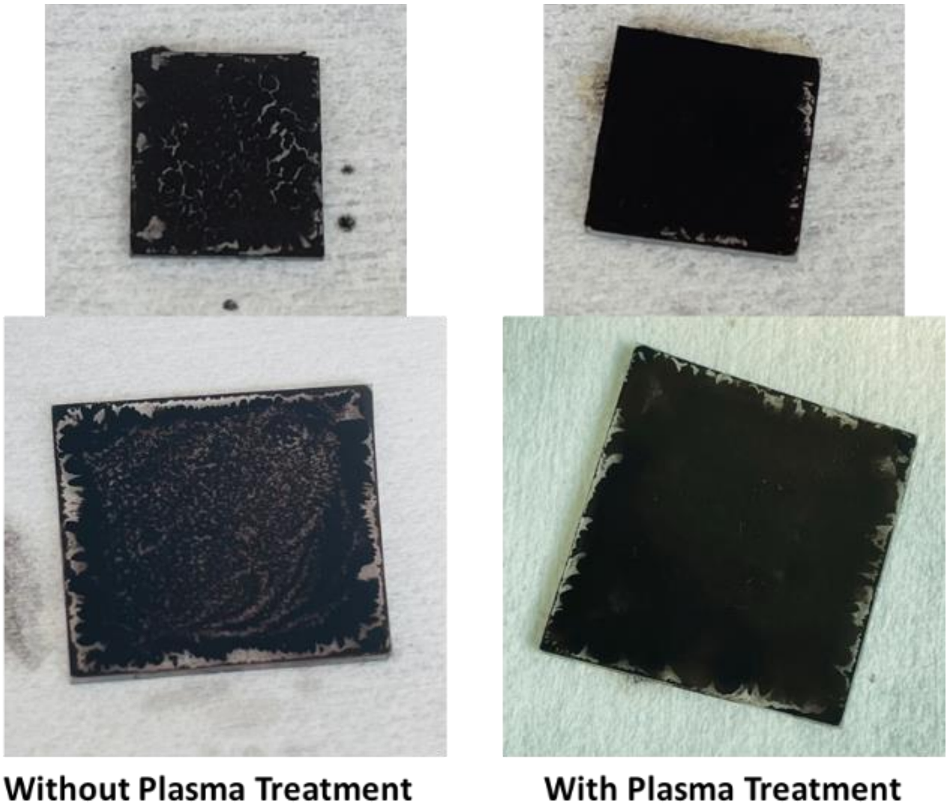

Figure S15. Synthesized CMP (**BI** and **PI**) drop-casted on 1×1 cm^2 pieces of thermal SiO_2 and 3×3 cm^2 fused silica pieces without and with the plasma treatment.

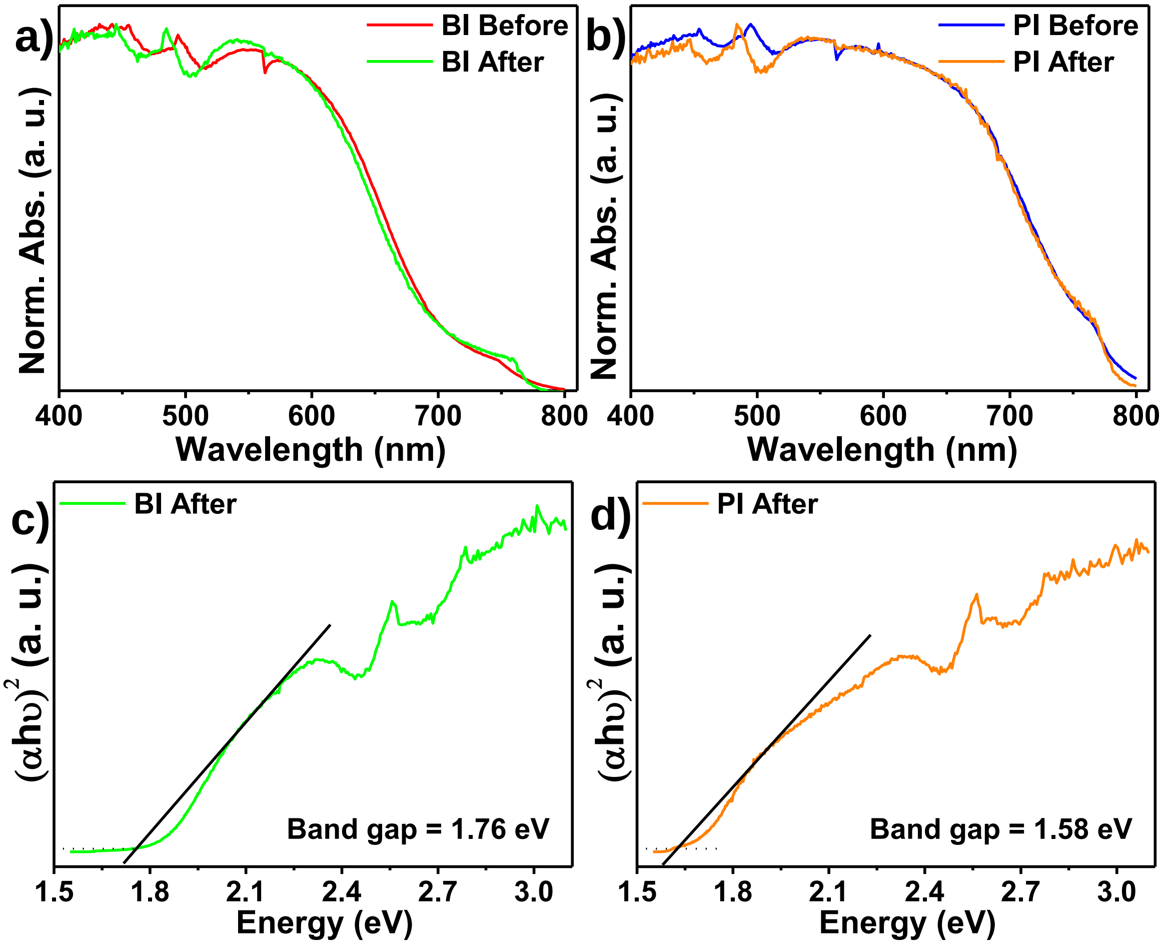

Figure S16. Comparison of UV/VIS absorption spectra of a) **BI** and b) **PI** in the solid state before and after oxygen plasma-treatment assisted thin film preparation. Tauc plot of c) **BI** and d) **PI** for bandgap calculations after oxygen plasma treatment-assisted thin film preparation.

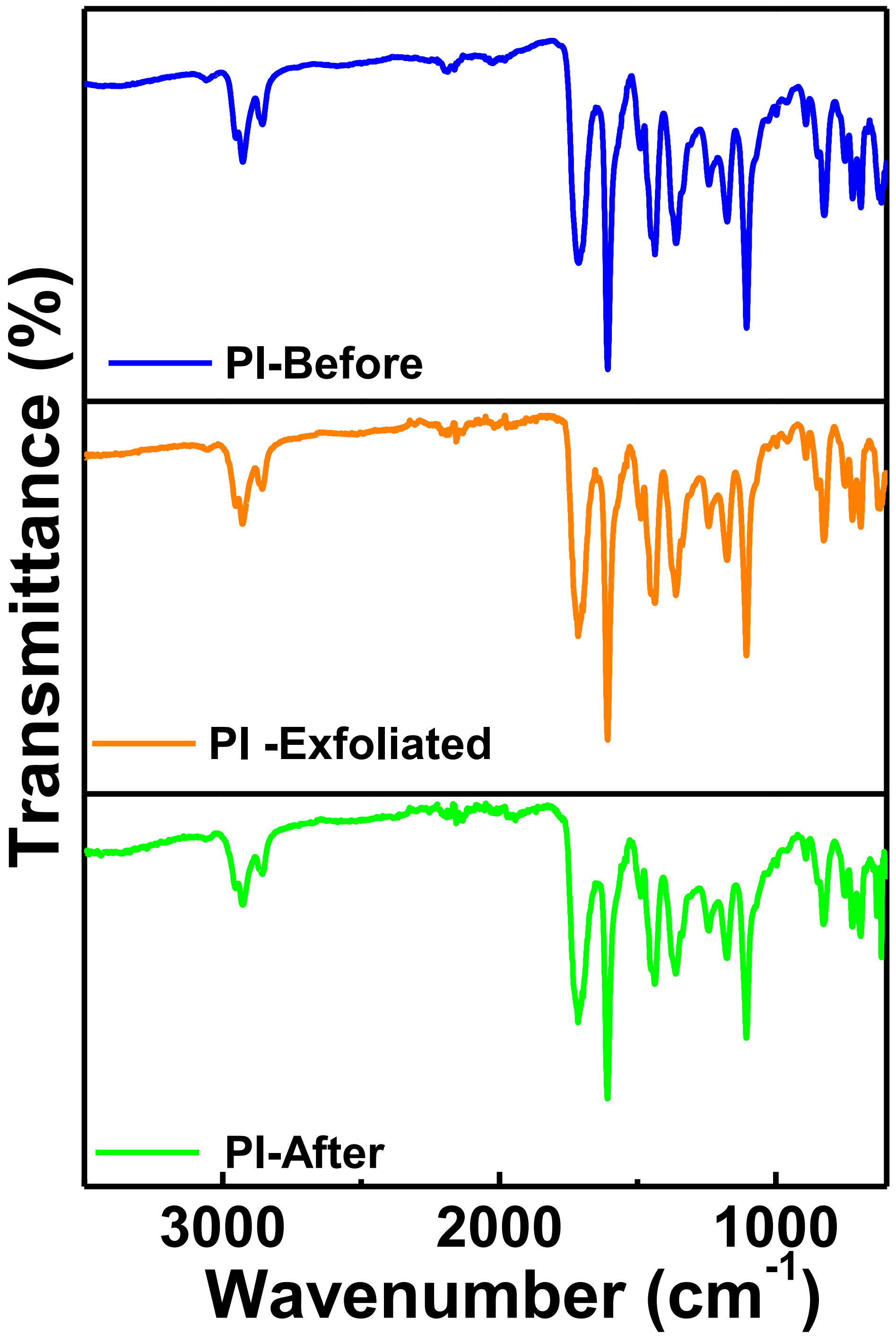

Figure S17. Comparison of FT-IR spectra of **BI** and **PI** pristine (top panel), after exfoliation (middle panel), after oxygen plasma-treatment assisted thin film preparation (bottom panel).

Table S2. Conductivity and mobility of **BI** and **PI** thin films relative to recent literature reports.

Name	Type	Method	Conductivity (mS/cm)	Mobility (cm^2/Vs)	Ref.
**CuPc-pz**	p-type 2D-COF	Hall effect	3.3×10^{-4}	0.9 ± 0.2	[14]
**ZnPc-pz**	p-type 2D-COF	Hall effect	7×10^{-4}	4.8 ± 0.7	
**BUCT-COF-1**	n-type 3D-COF	Hall effect	1.6×10^{-4}	2.74 ± 0.2	[15]
**HP-FAN**	p-type 2D-COF	Hall effect	38.2	1.49	[16]
		Field-effect	-	1.42	
**PCIBT**	p-type 2D-CMP	Field-effect	-	1.7	[17]
**PCITV**	p-type 2D-CMP	Field-effect	-	0.39	
**PI**	n-type 2D-CMP	Hall effect	5.31	6.6 ± 0.22	**This Work**
**BI**	n-type 2D-CMP	Hall effect	0.17	3.5 ± 0.96	

6. References

1. a) J. Mei, K. R. Graham, R. Stalder, J. R. Reynolds, *Org. Lett.* 2010, 12, 4, 660-663; b) T. Skorjanc, Dinesh Shetty, M. E. Mahmoud, F. Gándara, J. I. Martinez, A.K. Mohammed, S. Boutros, A. Merhi, E. O. Shehayeb, C.A. Sharabati, P. Damacet, J. Raya, S. Gardonio, M. Hmadeh, B. R. Kaafarani, A. Trabolsi, *ACS Appl. Mater. Interfaces* **2022**, *14*, 2015-2022.

2. J. Bardeen, W. Shockley, *Phys. Rev.* **1950**, *80*, 72.

3. F. B. Beleznay, F. Bogár, J. Ladik, *J. Chem. Phys.* **2003**, *119*, 5690-5695.

4. J. Xi, M. Long, L.Tang, D. Wang, Z.Shuai, *Nanoscale*, **2012**,*4*, 4348-4369.

5. Y. K. Chung, J. Lee, W.-G. Lee, D. Sung, S. Chae, S. Oh, K. H. Choi, B. J. Kim, J.-Y. Choi, J. Huh, *ACS Omega*, **2021**, *6*, 26782-26790.

6. Frisch, M. J. *et al.*, Gaussian 16 Rev. C.01, Wallingford, CT, **2016**.

7. T. Yanai, D. P. Tew, N. C. Handy , *Chem. Phys. Lett.* **2004**, 393, 51-57.

8. T. H. Dunning, Jr. *J. Chem. Phys.* **1989**, *90*, 1007-1023.

9. P. Giannozzi, S. Baroni, N. Bonini, M.Calandra, R.Car, C. Cavazzoni, D. Ceresoli, G. L Chiarotti, M. Cococcioni, I. Dabo, *J. Phys.: Cond. Matter*, **2009**, *21*, 395502.

10. J. P. Perdew, K. Burke, M. Ernzerhof, Phys. Rev. Lett. **1996**, *77*, 3865.

11. S. Grimme, *J. Comput. Chem.* **2006**, *27*, 1787-1799.

12. A. M. Rappe, K. M. Rabe, E. Kaxiras, J. D. Joannopoulos, *Phys. Rev. B*, **1990**, *41*, 1227.

13. J. D. Pack, H. J. Monkhorst, *Phys. Rev. B*, **1977**, 16, 1748.

14. M. Wang, M. Ballabio, M. Wang, H.-H. Lin, B. P. Biswal, X. Han, S. Paasch, E. Brunner, P. Liu, M. Chen, M. Bonn, T. Heine, S. Zhou, E. Cánovas, R. Dong, X. Feng, *J. Am. Chem. Soc.* **2019**, *141*, 16810-16816.

15. S. Wang, L. Da, J. Hao, J. Li, M. Wang, Y. Huang, Z. Li, Z. Liu, D. Cao *Angew. Chem.* **2021**, *133*, 9407-9411.

16. H.-J. Noh, S.Chung, M. S. Okyay, Y.-K. Im, S.-W. Kim, D.-H. Kweon, J.-P. Jeon, J.-M. Seo, N.-H. Kim, S.-Y. Yu, Y. Reo, Y.-Y. Noh, B. Kang, N. Park, J. Mahmood, K. Cho, J.-B. Baek., *Chem*, **2022**, *8*, 3130-3144.

17. J.-J. Park, Y.-A. Kim, S.-H. Lee, J. Kim, Y. Kim, D.-H. Lim, D.-Y. Kim, *ACS Appl. Polym. Mater.* **2019**, *1*, 27–35.
